# MliR, a novel MerR-like regulator of iron homeostasis, impacts metabolism, membrane remodeling, and cell adhesion in the marine Bacteroidetes *Bizionia argentinensis*

**DOI:** 10.3389/fmicb.2022.987756

**Published:** 2022-09-02

**Authors:** Leonardo Pellizza, Magalí G. Bialer, Rodrigo Sieira, Martín Aran

**Affiliations:** Fundación Instituto Leloir and Instituto de Investigaciones Bioquímicas de Buenos Aires (IIBBA-CONICET), Buenos Aires, Argentina

**Keywords:** iron metabolism, transcriptional regulation, Bacteroidetes, filamentation, iron uptake, energy metabolism

## Abstract

The MerR family is a group of transcriptional activators with conserved N-terminal helix-turn-helix DNA binding domains and variable C-terminal effector binding regions. In most MerR proteins the effector binding domain (EBD) contains a cysteine center suited for metal binding and mediates the response to environmental stimuli, such as oxidative stress, heavy metals or antibiotics. We here present a novel transcriptional regulator classified in the MerR superfamily that lacks an EBD domain and has neither conserved metal binding sites nor cysteine residues. This regulator from the psychrotolerant bacteria *Bizionia argentinensis* JUB59 is involved in iron homeostasis and was named MliR (MerR-like iron responsive Regulator). *In silico* analysis revealed that homologs of the MliR protein are widely distributed among different bacterial species. Deletion of the *mliR* gene led to decreased cell growth, increased cell adhesion and filamentation. Genome-wide transcriptomic analysis showed that genes associated with iron homeostasis were downregulated in *mliR*-deletion mutant. Through nuclear magnetic resonance-based metabolomics, ICP-MS, fluorescence microscopy and biochemical analysis we evaluated metabolic and phenotypic changes associated with *mliR* deletion. This work provides the first evidence of a MerR-family regulator involved in iron homeostasis and contributes to expanding our current knowledge on relevant metabolic pathways and cell remodeling mechanisms underlying in the adaptive response to iron availability in bacteria.

## Introduction

The MerR family of transcriptional regulators are dimeric proteins with an N-terminal helix-turn-helix DNA binding domain (DBD), followed by an antiparallel coiled-coil subunit interaction region, and usually by a C-terminal effector binding domain (EBD). This family is distinguished by the high conservation in the DBD and low aminoacid similarity in the EBD, consistent with the role of this domain in each protein in sensing effector molecules. Most members of the family are activators and act at promoters with unusually long spacer of 19 bp (Hobman, 2007) in response to the binding of inducers by distorting the promoter DNA to allow open complex formation and transcriptional activation (Brown et al., 2003).

The prototype for the MerR family of transcription factors is the regulator of the mercury resistance (mer) operon found on the transposable elements Tn21 and Tn501 in Gram-negative bacteria (Brown et al., 1983, 1986). Subsequently, proteins in the MerR family present in a number of bacterial species were shown to share common structural features (Brown et al., 2003). In MerR proteins the EBD contains a metal binding pocket formed by three cysteines. One Cys arises from one monomer (Cys82 in *Escherichia coli* MerR) and two Cys come from the other monomer (Cys117 and Cys126 in *E. coli* MerR) (Utschig et al., 1995). In MerR the Cys center binds to Hg (II), but other members of the family can bind other metals like Pb (PbrR), Cu (CueR), and Zn (ZntR). In addition, the Cys center in the oxidative stress sensor SoxR (Amabile-Cuevas and Demple, 1991) binds to a [2Fe–2S] cluster to sense superoxide concentrations.

Over the last decade, however, it has become clear that the MerR family of regulators is more diverse than originally recognized. Other MerR-like regulators have no Cys centers in their EBD regions and are able to sense multiple compounds with diverse chemical properties (Holmes et al., 1993; Ahmed et al., 1995; Baranova et al., 1999), or even light (Takano et al., 2011). Furthermore, transcription factors initially placed in the MerR family were subsequently classified in other DNA-binding protein families. Particularly, TnrA and GlnR appear to have evolved from or represent a separate branch of the MerR family and are founding members of a new family of transcription regulators that control *B. subtilis* nitrogen homeostasis (Schumacher et al., 2015). The TnrA/GlnR and MerR proteins have similar DBD motifs, but their modes of interaction and assembly are completely different. The availability of completed bacterial genome sequences and the advent of new structural tools (Anonymous, 2021) have enabled us to search for the presence of additional types of MerR-like regulators.

The Bacteroidetes is a vast phylum with diversity at every level of resolution, from the genus down to the genomes of strains (Johnson et al., 2017). These bacteria are all Gram negative, and cover a mixture of physiological types, from strictly anaerobic *Bacteroides* to strictly aerobic *Flavobacteria* (Bernardet and Nakagawa, 2006). Outstanding characteristics of Bacteroidetes are their gliding motility (McBride, 2019), their ability to degrade and absorb complex biopolymers given the large number of peptidases, glycosidases and TonB-dependent outer membrane receptors/transporters (Terrapon et al., 2015), as well as the use of pigments such as flexirubin and carotenoids as UV protectors (Vila et al., 2019). Members of the Bacteroidetes have colonized virtually all types of habitats on Earth. They are among the major members of the microbiota of animals, especially in the gastrointestinal tract, can act as pathogens and are frequently found in soils, oceans and freshwater. Environmental Bacteroidetes represent a key compartment for carbon fluxes and budgets in ecosystems. In these contrasting ecological niches, Bacteroidetes are increasingly regarded as specialists for the degradation of high molecular weight organic matter (Thomas et al., 2011b).

The majority of regulators in the MerR superfamily respond to environmental stimuli, such as oxidative stress, heavy metals or antibiotics (Brown et al., 2003). Bacteroidetes, on the other hand, exhibit considerable nutritional flexibility and have the ability to respond to diverse environmental stresses. Therefore, these organisms represent valuable sources to expand our current knowledge on the diversity of MerR superfamily regulators.

In this context, we investigated the MerR-like regulators present in the psychrotolerant bacteria *Bizionia argentinensis* JUB59. Members of the genus *Bizionia* have been isolated from diverse marine environments including the intestinal tract of marine vertebrates and invertebrates (Nedashkovskaya et al., 2010; Kim et al., 2015, 2018). *Bizionia argentinensis* JUB59 was isolated from surface seawater in Antarctica (Bercovich et al., 2008) and it is included in the family *Flavobacteriaceae*, the largest family of the *Bacteroidetes* phylum. We have previously characterized the function of novel proteins present in *B. argentinensis* JUB59 that were largely conserved within the family *Flavobacteriaceae* (Smal et al., 2012; Aran et al., 2014; Pellizza et al., 2016, 2020; Cerutti et al., 2017). Here, we identified one putative regulator classified in the MerR superfamily of winged–HTH (helix–turn–helix)–coiled-coil DNA-binding proteins but lacking an EBD. We show that this novel MerR-like transcription factor is involved in iron homeostasis and was named MliR (MerR-like iron responsive Regulator). In contrast to most MerR proteins, which contain EBD domains with conserved cysteine residues, MliR has neither conserved metal binding sites nor cysteine residues. The structural comparison of MliR with representatives of the TnrA/GlnR and MerR protein families revealed similar winged–HTH regions, but some remarkable differences in its N-terminal and C-terminal domains. We revealed that homologs of the MliR protein are widely distributed among different bacterial species. Deletion of the *mliR* gene led to decreased cell growth, increased cell adhesion and filamentation. RNA sequencing analysis showed that expression of genes related to iron homeostasis, transmembrane transport, metal ion transport, cellular amino acid metabolism and iron-sulfur cluster assembly, was downregulated in *mliR*-deletion mutant. Through flow cytometry, Nuclear magnetic resonance (NMR)-based metabolomics, ICP-MS, fluorescence microscopy and biochemical analysis we evaluated metabolic and phenotypic changes associated with *mliR* deletion. This work provides the first evidence of a MerR-family regulator involved in iron homeostasis and contributes to expanding our current knowledge on iron metabolism in bacteria.

## Results

### MliR homologs are widely distributed in bacteria and share protein domains with members of the MerR and TnrA/GlnR protein families

**FIGURE 1:**
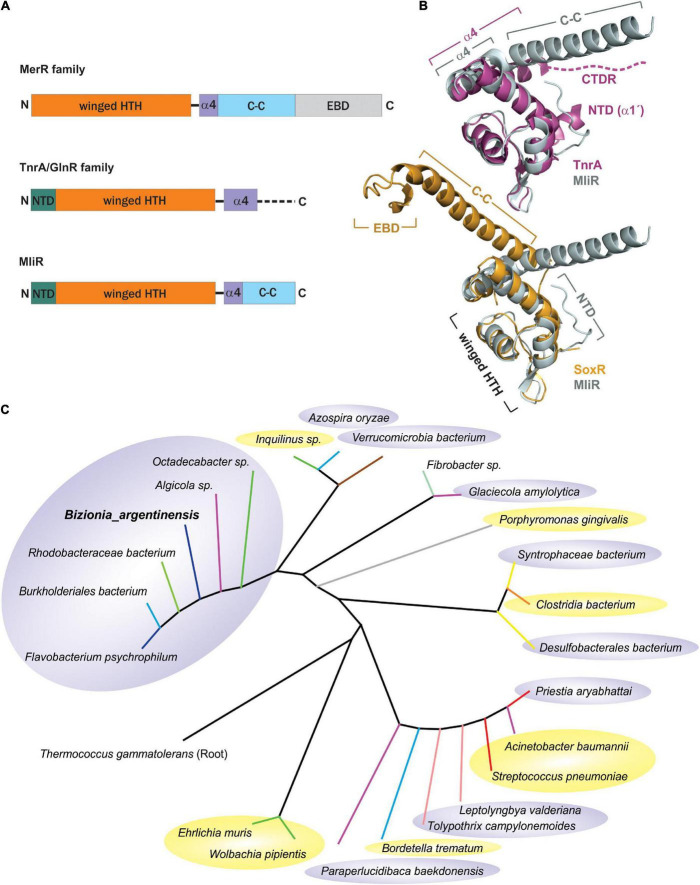
Taxonomic distribution and structural comparison of MliR with members of the MerR and TnrA/GlnR protein families. **(A)** Domain architecture of the MliR protein and representative proteins of the MerR and TnrA/GlnR families. The winged–HTH DNA binding domain (orange), the alpha helix α4 motif (purple), the antiparallel coiled-coil domain (C-C, light blue rectangle), the EBD domain (light gray) and the NTD (green) are represented. The dashed line indicates the disordered C-terminal domain. **(B)** Structural comparison of MliR with representatives of the TnrA/GlnR and MerR protein families. The superposed 3D structures of MliR (Alpha fold model, light gray) with TnrA (PDB ID: 4R22, purple) and SoxR protein (PDB ID: 2ZHG, light orange) are shown. Relevant protein domains and motifs described in A are indicated. The dashed line represents the C-terminal disordered region (CTDR) in TnrA. **(C)** Phylogenetic analysis of MliR homologous sequences from diverse bacteria species. The rooted phylogenetic tree was inferred using the Maximum likelihood method and the JTT matrix-based model. Tree branches are colored according to bacteria class as follows: Flavobacteriia (blue), Bacteroidia (gray), Betaproteobacteria (light blue), Alphaproteobacteria (green), Gammaproteobacteria (purple), Verrucomicrobiae (brown), Bacilli (red), Clostridia (orange), Fibrobacteria (light green), Deltaproteobacteria (yellow), Cyanophyceae (light pink). Pathogenic bacteria species are highlighted in yellow ellipses. Environmental non-pathogenic bacteria species are highlighted in gray ellipses.

Members of the MerR superfamily of transcriptional regulators are conserved across bacterial species (Brown et al., 2003). In particular, the *B. argentinensis* JUB59 genome contains one putative regulator in this superfamily, the *BZARG_RS02055* gene codified in contig 3 (327 nt, 109 residues). The amino acid sequence of the predicted DNA-binding domain of *BZARG_RS02055* (MliR from here on) has resulted in its placement in the MerR superfamily of winged–HTH (helix–turn–helix)–coiled-coil DNA-binding proteins (Figure 1A) and is included in the MlrA-like sg2 subfamily from the NCBI’s CCD (Conserved Domain Database). However, unlike most MerR proteins, which contain EBD domains with conserved cysteine residues, MliR lacks an EBD domain and has neither conserved metal binding sites nor cysteine residues. In addition, it holds ∼11 extra N-terminal residues (N-Terminal domain, NTD), characteristic of the TnrA/GlnR protein family but notably absent in MerR proteins (Schumacher et al., 2015; Figure 1A). Consequently, given these distinctive features of MliR, it was challenging to predict its cellular function based solely on protein sequence information.

In order to *in silico* compare the structure of MliR with members of the MerR and TnrA/GlnR protein families, we modeled MliR of *B. argentinensis* JUB59 using the AlphaFold IA system (Jumper et al., 2021). The resulting model describes a conformation with the presence of the DNA-binding and coiled-coil domains (Figure 1B). The structural comparison of MliR with representatives of the TnrA/GlnR and MerR protein families (Schumacher et al., 2015) reveals similar winged–HTH regions, with a root mean squared deviation (RMSD) of 1.14 Å for 49 aligned C^α^ with TnrA (PDB ID: 4R22) and a RMSD of 1.26 Å for 44 aligned C^α^ with SoxR (PDB ID: 2ZHG), respectively (Figure 1B). However, MliR has some remarkable structural differences in comparison to members of the aforementioned protein families. The first 11 N-terminal residues in MliR are modeled as four short turns stabilized by a salt bridge (E15:K8), hydrophobic interactions (I3:F20; L5:A19; M1:F20) and hydrogen bonds (e.g., Y11:K8) (Supplementary Figure S1). In contrast, TnrA/GlnR family members harbor an N-terminal region composed by an alpha helix (α1’) with conserved hydrophobic residues crucial for dimer formation (Schumacher et al., 2015; Figure 1B). Further, although MliR presents some hydrophobic residues in its N-terminal region (e.g., M1, I3, L5), they do not appear to be conserved (neither in structure nor in sequence) within the TnrA/GlnR protein family. On the other hand, structural differences between MliR, TnrA/GlnR, and MerR family proteins are also notable in their C-terminal domains (Figure 1B). The α4 in MliR is similar to MerR proteins, spanning ∼9–11 residues, while α4 in TnrA/GlnR proteins is longer, spanning ∼18 residues. In particular, TnrA/GlnR proteins do not harbor a C-terminal coiled-coil dimerization motif, which is a hallmark of MerR proteins and also conserved in MliR. In fact, TnrA/GlnR C-terminal residues are disordered and thought to function as a sensor domain that folds only upon effector protein binding (Figure 1B; Schumacher et al., 2015).

Interestingly, through database searches of homologous protein sequences we found a considerable number of proteins categorized as “MerR-like transcriptional regulators” likely associated with MliR topology. These homologs share sequence identities between 30 and 70% with the *B. argentinensis* JUB59 MliR and their taxonomic distribution suggests that MliR homologs are present in a wide variety of environments (Figure 1C).

### Deletion of *mliR* affects cell growth, enhances cell adhesion and induces cell filamentation

The particular structural characteristics of MliR, coupled with the absence of an identified EBD, made it difficult to anticipate its cellular role. In order to address this issue, we implemented a gene deletion strategy in *B. argentinensis* JUB59. Despite the lack of genetic manipulation systems described for *B. argentinensis* JUB59, techniques to construct gene deletions in the chromosomes of several members of the Bacteroidetes have been established (Pumbwe et al., 2006; Rhodes et al., 2011; Wang et al., 2014; Zhu and McBride, 2014, 2016). In particular, we used the suicide vector pYT313 (Zhu et al., 2017), carrying PompA-sacB, to perform the gene deletion approach (Supplementary Figure S2). After a second recombination event, cells we plated on agar medium containing sucrose to select a clone lacking the wild-type gene. Of the nine analyzed sucrose-resistant colonies, five were *mliR* deletion mutants and the remaining four were wild type for the *mliR* locus (Supplementary Figure S2).

**FIGURE 2:**
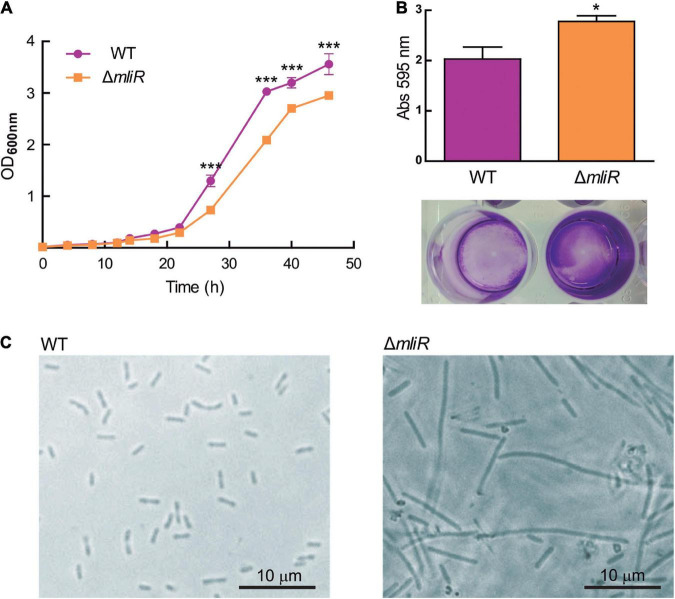
Growth curve, biofilm and cellular shape of WT and Δ*mliR* mutant *B. argentinensis* JUB59. **(A)** The growth curves of WT (purple) and Δ*mliR* mutant (orange) *B. argentinensis* JUB59 are shown. Cells were grown in Marine Broth at 22°C. The mean OD600 ± SD from three individual experiments is plotted. **(B)** Crystal violet staining and quantification of WT (purple) and Δ*mliR* mutant (orange) *B. argentinensis* JUB59 biofilms. Quantification was carried out by absorbance at 575 nm and values were normalized to dry weight. The mean OD575 ± SD from three individual experiments is plotted. **(C)** Phase-contrast microscopy of WT and Δ*mliR B. argentinensis* JUB59 cultures. In **(A,B)** the statistical significance was assessed by Student’s *t*-test. *p < 0.05; ***p < 0.001.

To explore the influence of *mliR* deletion, the growth curve, the biofilm formation capacity, and the cellular shape were evaluated in the parent strain (WT, *B. argentinensis* JUB59) and the Δ*mliR* mutant strain. The WT strain showed a typical growth curve, with a relatively long lag phase (0–20 h), followed by an exponential phase, during which major bacterial growth occurred (20–33 h), and then a stationary phase (Figure 2A). In contrast, Δ*mliR* strain grew more slowly after 15 h, particularly between 22 and 45 h. During this phase, the OD600 values of Δ*mliR* strain were significantly lower than those of the WT strain (Figure 2A). In addition, Δ*mliR* cells showed a significantly higher degree of biofilm formation in comparison to WT cells as assessed with a standard method (crystal violet analysis) (Figure 2B). But more interestingly, microscopic images of Δ*mliR* cells revealed a remarkable difference in morphology with respect to WT cells (Figure 2C). The Δ*mliR* cells appeared as elongated filaments (3–30 μm), while WT cells showed a typical rod shape (1.5–3 μm), as previously reported (Bercovich et al., 2008).

**FIGURE 3:**
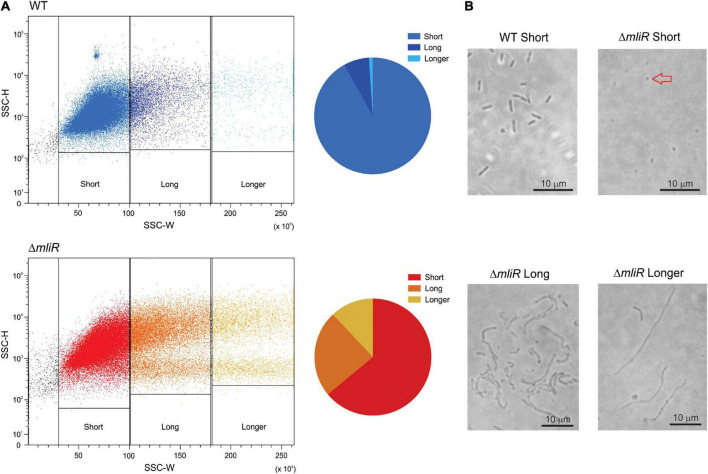
Flow cytometry and microscopic analysis of WT and Δ*mliR B. argentinensis* JUB59 populations of varying cell lengths. **(A)** Flow cytometry analysis of the corresponding WT and Δ*mliR* populations are displayed as dot plots with SSC-H plotted against SSC-W. The cell length distributions are shown in pie charts for WT and Δ*mliR* populations. **(B)** Phase-contrast microscopy of WT and Δ*mliR* sorted cells populations. The red arrow points to a spherical cell.

In order to further characterize the differences in cell morphology between WT and Δ*mliR* cell populations, we implemented a flow cytometry approach that relies on the different light scattering properties of filamentous versus short cells, and does not require the use of fluorescent dyes (Burke et al., 2013). Using this method a trend of increasing side scatter width (SSC-W) signal with increasing cell length has been reported (Burke et al., 2013). The corresponding dot plots for WT and Δ*mliR* cell populations are shown, where each dot represents a single cell or event from each population (Figure 3). The populations of cells were sorted on the basis of increasing SSC-W (gates as shown in Figure 3). In line with the results described in Figure 2C, the distribution of cell lengths for Δ*mliR* strain showed a higher proportion of events with increased SSC-W values in comparison to the WT strain population (Figure 3). Sorted cells were subsequently examined using phase-contrast microscopy, which revealed that the populations from the gates with the smallest SSC-W values (“short”) were made up predominantly of non-filamentous cells of less than 3 μm in length. Surprisingly, however, while the WT cells showed the typical rod shape, the Δ*mliR* cells were found to be spherical, similar to cocci, with an average size between 0.75 and 1 μm (Figure 3). In addition, populations of Δ*mliR* cells sorted from gates with increasing SSC-W values (“long” and “longer”) were enriched for filamentous cells (Figure 3). Interestingly of note, Δ*mliR* cells from the “long” population were generally located in clusters in the optical field, which may be related to their higher tendency to biofilm formation with respect to WT cells. In contrast, we were unable to identify cells from the “long” and “longer” populations of the WT strain, probably because of their low concentration and wide dispersion in the optical field.

**FIGURE 4:**
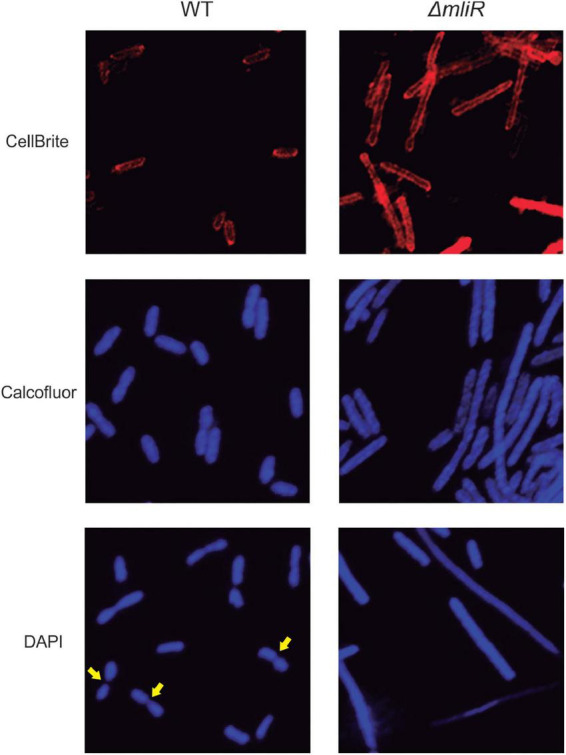
Fluorescence microscopy analysis of WT and Δ*mliR B. argentinensis* JUB59 cells. Exponentially growing cultures were stained with the membrane dye CellBrite or Calcofluor White Stain. The cells incubated with CellBrite were then fixed and stained with DAPI. Representative micrographs for WT and Δ*mliR* cells are shown. Yellow arrows highlight cell septum.

Filamentation morphology is a cryptic phenomenon which consists of an alteration or lack of cell septation during the cell growth, as a consequence of DNA damage or development of stress, such as nutritional factors, antibiotic resistance and low temperature, among many others (Ultee et al., 2019; Rizzo et al., 2020). In this context, with the aim of exploring the morphology and division pattern of WT and Δ*mliR* cells, we performed a fluorescence microscopic analysis. To this end, exponentially growing cultures were stained with the membrane dye CellBrite or Calcofluor White Stain, a polysaccharide-binding dye (Luna et al., 1995). The cells incubated with CellBrite were then fixed and stained with DAPI. Representative micrographs for WT and Δ*mliR* cells are shown in Figure 4. WT cells displayed a division phenotype mainly formed by two cell units per chain, as previously observed (Figure 2C). This was clearly evidenced with DAPI staining. However, filaments from Δ*mliR* cells did not show regular spaced septa neither visible with DAPI nor with membrane stains. The lack of observable constrictions in Δ*mliR* cells may indicate that outer membrane invagination is diminished in this mutant. Nevertheless, through a detailed inspection of micrographs, we detected a small population of Δ*mliR* filaments with constrictions at one of their poles (Supplementary Figure S3). The shape and the size of the small daughter cells from those filaments were strikingly similar to those of the sorted Δ*mliR* “short” cells (Figure 3).

### Global transcriptional analysis reveals downregulation of iron homeostasis and metal transport-related genes in the *mliR*-deletion mutant

In order to assess a global view of the regulatory impact of MliR in *B. argentinensis* JUB59, we conducted whole-transcriptome shotgun sequencing (RNA-seq) to compare the transcriptomes of the WT and the Δ*mliR* strains grown on marine broth. Total RNA was prepared from bacterial cells harvested at exponential phase. The mRNA fractions were then subjected to a differential expression analysis. As a result, 49 transcripts (excluding hypothetical proteins) with statistically significant differential expression changes were detected in the Δ*mliR* strain compared to the WT strain (log_2_-fold change > 1 and adjusted *P*-value < 0.05) (Supplementary Table S3). In particular, the largest differences were observed for 10 transcripts (log_2_-fold change > 2), which were found to be downregulated in the Δ*mliR* strain, suggesting a regulatory role of MliR as a transcriptional activator. These transcripts included genes encoding a siderophore uptake receptor (TonB-dependent); two electron transfer proteins [Rieske (2Fe–2S) and Cytochrome c]; a D-Serine dehydratase, that catalyze the degradation of D-Ser to pyruvate and ammonia; an AraC binding domain containing protein; a superoxide dismutase; an asparagine synthetase B; an imelysin-like protein and a FeoB-associated Cys-rich membrane protein, part of a Fe^2+^ uptake system. In contrast, only one transcript encoding a DNA polymerase IV with log_2_-fold change < –2 was found to be upregulated in the Δ*mliR.*

**FIGURE 5:**
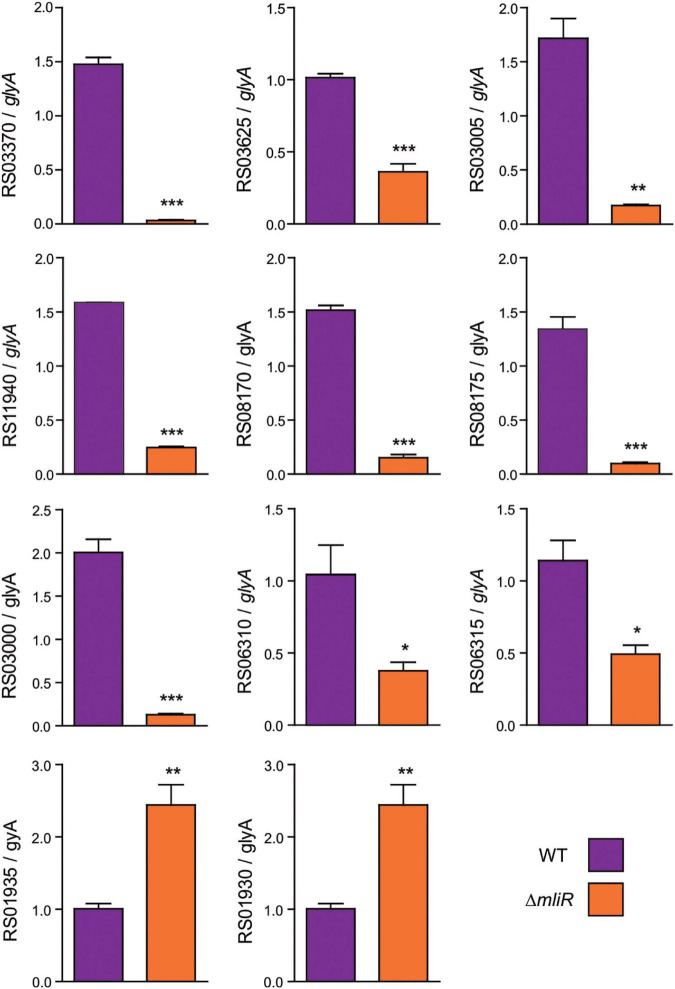
RT-qPCR of selected differentially expressed genes identified in the transcriptome analysis. Total cDNA from WT and Δ*mliR B. argentinensis* JUB59 was prepared. Transcript levels were measured by RT-qPCR using cDNAs as template and the primers listed in Supplementary Table S1. Transcript levels were normalized to that of the serine hydroxymethyltransferase A (*glyA*) housekeeping. The statistical significance was assessed by Student’s *t*-test. *p < 0.05; **p < 0.01; ***p < 0.001. *BZARG_RS03625*: TonB-dependent receptor; *BZARG_RS03370*: TonB-dependent receptor; *BZARG_RS03005*: Rieske (2Fe–2S) protein; *BZARG_RS11940*: Superoxide dismutase; *BZARG_RS08175*: D-serine ammonia-lyase; *BZARG_RS08170*: AraC binding Domain; *BZARG_RS03000*: Cytochrome c; *BZARG_RS06310*: Mn^2+^/Zn^2+^ ABC transporter; *BZARG_RS06315*: Zn^2+^ ABC transporter; *BZARG_RS01930*: DNA polymerase IV; *BZARG_RS01935*: DNA polymerase III subunit alpha.

To confirm the differentially expressed genes (DEGs) identified in our transcriptome analysis, 10 randomly selected DEGs were evaluated by Reverse transcription quantitative real-time PCR (RT-qPCR). The RT-qPCR results showed that 8 down-regulated DEGs [such as TonB-dependent receptors, ATP binding cassette (ABC) transporters and Cytochrome *c*] demonstrated higher expression levels in WT than in Δ*mliR* (Figure 5). The two up-regulated DEGs (DNA polymerase III subunit alpha and DNA polymerase IV, involved in DNA repair) showed lower expression levels in WT than in Δ*mliR*. Expression trends were consistent for all transcripts in RT-qPCR and RNA-seq analyses.

To assess the possible functional significance associated with MliR activity, we performed an enrichment analysis of the differential expression data using the FunRich tool (Fonseka et al., 2021). The analysis was focused on genes that displayed log_2_-fold change values higher than 1 and presented annotated data on its molecular function, biological process or sub-cellular localization at the Uniprot database. As shown in Supplementary Figure S6, most of the downregulated genes in Δ*mliR* strain encoded proteins involved in iron homeostasis, transmembrane transport and metal ion transport. These three biological processes exhibited the highest enrichment within the downregulated transcripts. The genes classified in these categories included two TonB-dependent receptors (siderophore uptake), a TonB energy transducer, four metal ion ABC transporters (for Fe^+3^, Mn^2+^, and Zn^2+^) and three proteins involved in Fe^2+^ uptake. In addition, biological processes associated with oxidative stress, cellular amino acid metabolism and iron-sulfur cluster assembly showed a lower degree of enrichment.

On the other hand, the upregulated genes in Δ*mliR* strain showing the highest enrichment mainly encoded proteins associated with translation, DNA repair/replication and RNA processing. A lesser extent of enrichment, however, was observed for efflux transmembrane transport, lipid metabolic process and intracellular signal transduction.

### The *mliR*-deletion mutant is deficient in iron, manganese, and zinc uptake

**FIGURE 6:**
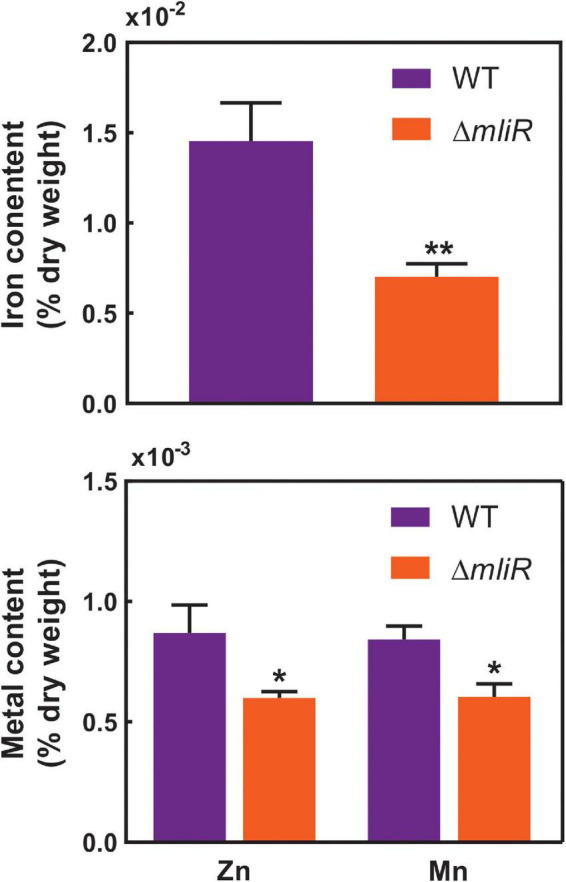
Intracellular concentration of metal ions in WT and Δ*mliR* cells. Metal ion concentration was determined by inductively coupled plasma mass spectrometry (ICP-MS). Metal content is expressed as % of dry weight. The statistical significance was assessed by Student’s *t*-test. *p < 0.05; **p < 0.001.

Based on our results showing downregulation of iron homeostasis and metal transport-related genes in the *mliR*-deletion mutant (Figure 5 and Supplementary Figure S6), we wondered if this deficiency could in fact alter its intracellular metal concentration. To address this issue, we used inductively coupled plasma mass spectrometry (ICP-MS) to compare the intracellular concentration of iron, manganese and zinc of WT and Δ*mliR* cells harvested at exponential phase. As depicted in Figure 6, Δ*mliR* cells showed significantly lower levels of iron, manganese and zinc than WT cells, thus revealing a clear correlation between altered ion transport processes and a metal deficiency phenotype in the *mliR*-deletion mutant.

### The expression of *mliR* is modulated in response to the extracellular concentration of ferric citrate in WT *Bizionia argentinensis* JUB59

**FIGURE 7:**
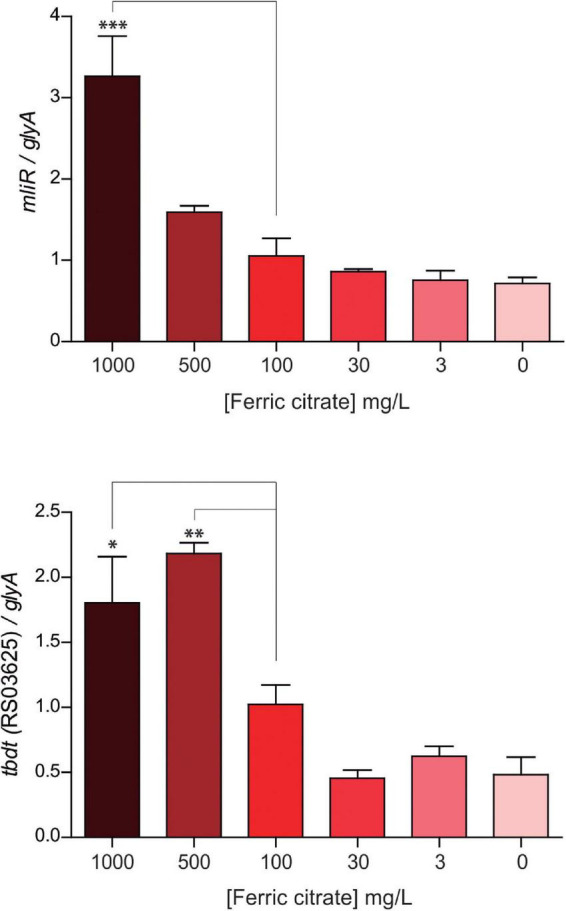
Expression of *mliR* as a function of the extracellular concentration of ferric citrate. WT *B. argentinensis* JUB59 was gown in iron-free Marine Broth 2216 supplemented with various concentrations of ferric citrate, as indicated. The suspensions were incubated for 4 h at 22°C. Total cDNA was prepared and transcripts levels were measured by RT-qPCR. Transcript levels of *mliR* and *tbdt* (RS03625) were normalized to that of the serine hydroxymethyltransferase A (*glyA*) housekeeping. The statistical significance was assessed by Student’s *t*-test. *p < 0.05; ^**^p < 0.01; ^***^p < 0.001.

By means of a global transcriptional analysis of the WT and the Δ*mliR B. argentinensis* JUB59 strains we revealed that MliR was associated with the upregulation of iron uptake-related proteins, leading to the idea that MliR could respond to extracellular iron availability and may play a role in the homeostasis of this metal. To investigate this issue, we determined by RT-qPCR the mRNA levels of the *mliR* gene in WT cells after four hours exposure to varying ferric citrate concentrations (0 mg/l up to 1000 mg/l). The *mliR* transcripts showed a significantly threefold enhancement when ferric citrate was increased from 0 mg/l to 1000 mg/l (Figure 7). This result is consistent with the notion that MliR is a transcriptional regulator whose expression is modulated by the extracellular concentration of iron.

Additionally, we examined the expression of a TonB-dependent receptor (*BZARG_RS03625*), one of the highly downregulated transcripts in Δ*mliR* strain and thought to be involved in iron uptake through siderophores. Notably, the *BZARG_RS03625* transcript levels displayed a significant twofold increment when ferric citrate was increased from 0 mg/l to 500 mg/l, thus showing a similar behavior to *mliR* transcripts (Figure 7). This observation is in agreement with our results in RNA-seq analysis and reinforces the idea that MliR could act as a positive transcriptional regulator.

### Nuclear magnetic resonance-based metabolomics shows global metabolic changes and impaired 3-deoxy-manno-octulosonate-8-phosphate (Kdo-8-P) production in *mliR*-deletion mutant

**FIGURE 8:**
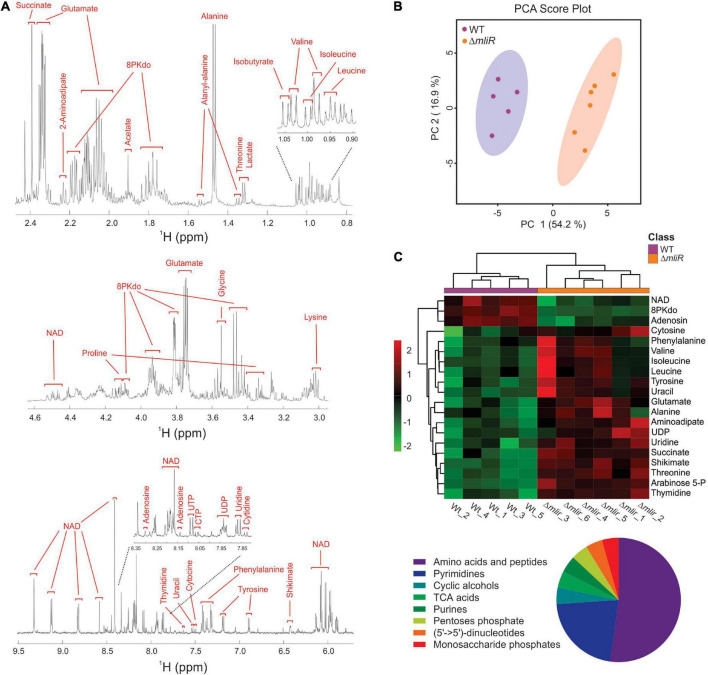
Untargeted ^1^H-NMR metabolomics of *B. argentinensis* JUB59. **(A)** Typical 600 MHz ^1^H-NMR spectrum of WT cells. Assigned resonances of specific metabolites are indicated in red. **(B)** PCA score plot derived from the 600 MHz ^1^H NMR spectra of WT (purple dots) and Δ*mliR* (orange dots) samples. **(C)** Multivariate hierarchical cluster analysis of significant differentially metabolites between WT (purple) and Δ*mliR* (orange) samples. The proportions of enriched class of significant differentially metabolites are depicted below in a pie chart.

To identify metabolic changes in the *mliR*-deletion mutant, we carried out untargeted ^1^H-NMR metabolomics on methanol-extracted samples from bacterial cells grown in marine broth and harvested at exponential phase. A total of 32 compounds were detected and identified in WT and Δ*mliR* cells, including most amino acids, carboxylic acids, and nucleotides (Figure 8 and Supplementary Table S4). A principal component analysis (PCA) and a partial least square-discriminant analysis (PLS-DA) were performed in order to detect the differences among WT and Δ*mliR* cells (Figure 8B and Supplementary Figure S4). In the PCA, the two first principal components (PC) explained more than 70% of the total variation, giving a clearer separation between groups.

In order to analyze the variation for each identified metabolite and visualize general grouping information, an unsupervised multivariate hierarchical cluster analysis (HCA) was performed (Figure 8C and Supplementary Table S5). The dendrograms grouped compounds into two clusters, where a large proportion of metabolites were increased in Δ*mliR* cells, mainly amino acids, pyrimidine nucleotides and organic acids.

From our global transcriptional analysis we found that *Rieske* and *Cytochrome c* transcripts were significantly downregulated in Δ*mliR* cells (Supplementary Table S3). Rieske is an iron–sulfur protein component of the cytochrome bc1 complex in bacteria (respiratory protein complex III) (Rieske et al., 1964; Kaila and Wikstrom, 2021). Cytochrome c is a small protein that functions as an electron carrier between respiratory protein complexes III and IV (Kaila and Wikstrom, 2021). Consequently, the electron flow of the respiratory chain is expected to be diminished in the Δ*mliR* mutant. For instance, the activity of the succinate dehydrogenase (respiratory protein complex II), which reduces the membranous pool of ubiquinone via succinate, is also thought to be decreased. In line with this view, our metabolomics analysis clearly showed that succinate was markedly accumulated in the Δ*mliR* strain (Supplementary Figure S5). Accordingly, the activity of the tricarboxylic acids (TCA) cycle may be affected in the Δ*mliR* mutant.

Pool sizes of free amino acids represent the interplay between synthesis, degradation, and protein turnover. Most amino acids detected in our study were significantly accumulated in Δ*mliR* strain (i.e., Phe, Val, Ile, Leu, Tyr, Glu, Ala, and Thr) in comparison with WT strain (Figure 8C and Supplementary Figure S5). Additionally, shikimate, a substrate for the synthesis of aromatic amino acids, was significantly augmented in Δ*mliR* strain. Since several tRNAs were also found to be increased in Δ*mliR* strain with respect to WT strain (Supplementary Table S3), we hypothesize that higher levels of free amino acids in Δ*mliR* cells could be in part due to a decrease in *de novo* protein synthesis. Furthermore, the deficiency in the TCA cycle, a multiple entry point for amino acids, may contribute to the accumulation of free amino acids in the *mliR*-deletion mutant.

As an elemental redox carrier, NAD^+^ receives hydride from metabolic processes including glycolysis, the TCA cycle, and fatty acid oxidation to form NADH (Xie et al., 2020). As previously reported, *B. argentinensis* JUB59 is unable to perform glycolysis (Bercovich et al., 2008), thus TCA cycle and fatty acid oxidation are, in principle, the main NADH producing systems in this bacterium. As mentioned above, deletion of *mliR* would generate a deficiency in TCA, therefore, NAD^+^ utilization and NADH production would be also reduced. In this regard, it has been shown that NAD^+^ biosynthesis is controlled according to the nutritional status in bacterial cells (Santos et al., 2020). In line with this, we found that NAD^+^ levels were significantly lower in Δ*mliR* strain than in WT strain (Figure 8C and Supplementary Figure S5). Hence, our results are consistent with an energy restriction and a decreased NAD^+^ recycling capacity of Δ*mliR* cells with respect to WT cells.

Nucleotide biosynthesis is essential for bacterial life, with the end products involved in multiple cellular functions (Goncheva et al., 2022). In Particular, *de novo* pyrimidine biosynthesis ultimately produces thymine, uracil, and cytosine nucleotides, which are also modified and used for DNA and RNA replication (Goncheva et al., 2022). Our results clearly showed a significant increase in metabolites involved in the pyrimidine biosynthesis pathway (i.e., cytosine, uracil, thymidine, uridine, and UDP) in Δ*mliR* cells with respect to WT cells (Figure 8C and Supplementary Figure S5). We speculate that higher levels of pyrimidine nucleotides are indicative of reduced DNA and RNA replication in the *mliR*-deletion mutant.

Despite the fact that most amino acids were found to be significantly increased in Δ*mliR* cells, both lysine and aspartate were decreased as compared to WT cells (Supplementary Figure S5). Additionally, a significant accumulation of aminoadipate was observed in *mliR*-deletion strain (Figure 8C and Supplementary Figure S5). Interestingly, these three metabolites are involved in pathways of lysine synthesis (aspartate pathway) and degradation (saccharopine pathway) (Serrano et al., 2012). The reduced levels of aspartate and lysine together with aminoadipate accumulation suggest that lysine degradation rate is increased in Δ*mliR* strain with respect to WT strain. The activation of the lysine degradation pathway in Δ*mliR* could arise in response to a deficiency in the TCA cycle, in order to regenerate NAD^+^, and/or to an increase in siderophore biosynthesis, in response to a deficit of intracellular iron concentration (Burrell et al., 2012).

Strikingly, 3-deoxy-manno-octulosonate-8-phosphate (Kdo-8-P), showed significantly lower levels in the Δ*mliR* strain than in WT strain (Figure 8C and Supplementary Figure S5). Kdo-8-P is a precursor in the biosynthetic pathway of 3-deoxy-manno-octulosonate (Kdo) (Levin and Racker, 1959), which is the main component of the inner core of bacterial LPS. In bacteria, the biosynthetic pathway of Kdo-8-P has been well characterized and involves two enzymes; (i) D-arabinose-5-phosphate isomerase (KdsD), that converts D-ribulose-5-phosphate (Ru5P) into D-arabinose-5-phosphate (A5P), and (ii) Kdo-8-P synthase (KdsA), a metal-dependent enzyme that catalyzes the condensation of A5P and phosphoenol pyruvate (PEP) to generate Kdo-8-P (Smyth and Marchant, 2013). Interestingly, in contrast to Kdo-8-P, we found that A5P was significantly increased in Δ*mliR* cells as compared with WT cells (Figure 8C and Supplementary Figure S4). On the other hand, RNA-seq analysis showed no significant differences in *Ru5P* and *KdsA* mRNAs between Δ*mliR* and WT cells. Therefore, our results strongly suggest that the reaction catalyzed by KdsA could be inhibited in Δ*mliR* cells due to a cytoplasmic metal deficiency.

### *mliR*-deletion mutant shows altered lipopolysaccharide composition

**FIGURE 9:**
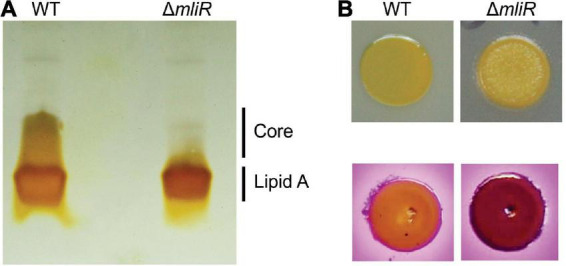
Lipopolysaccharide (LPS) structure of WT and *mliR*-deletion mutant *B. argentinensis* JUB59. **(A)** Outer membrane fractions were prepared and analyzed by SDS-PAGE after carbohydrate-specific periodate oxidation and silver staining. **(B)** Upper panel: surface colony appearance; lower panel: crystal violet-ammonium oxalate staining.

The outer membrane of Gram-negative bacteria is composed principally of lipopolysaccharide (LPS) and phospholipids. LPS has three main structural components: a hydrophobic anchor, lipid A; a complex oligosaccharide, core, further divided into inner core (IC) and outer core (OC); and an O-polysaccharide (OPS) chain composed of repeats (O units) of sugar residues (Guest et al., 2021). Kdo is an anionic eight-carbon sugar and is the central component of the inner core connecting the lipid A to the oligosaccharide chain. As shown above, Kdo-8-P is significantly reduced in *mliR* mutant, thereby altering the biosynthesis of Kdo. In this context, we wondered if this metabolic deficit could alter the LPS structure in Δ*mliR*. To this end, we analyzed the LPS compositions of WT and Δ*mliR* cells by periodate oxidation of carbohydrates followed by silver staining SDS-PAGE. The LPS profile of the WT strain showed the presence of one low-molecular-mass band assigned to the lipid A and a smeared band associated to the core region (Figure 9A). Since no carbohydrates-stained bands of slower mobility were detected, our results suggested that LPS molecules from WT *B. argentinensis* JUB59 were made up of lipid A and a core oligosaccharide. On the other hand, the LPS profile of the Δ*mliR* strain was composed of a single low-molecular-band attributed to the lipid A component while a band associated to the core region was undetectable (Figure 9A). This observation is in line with our metabolomics results and strongly suggests that the deficit in the synthesis of Kdo-8-P directly alters the WT architecture of the LPS in Δ*mliR* cells.

In order to investigate the impact of altered LPS in Δ*mliR* phenotype, we analyzed surface colony appearance and performed crystal violet-ammonium oxalate staining. The results showed that Δ*mliR* cells formed drier macrocolonies and presented higher crystal violet absorption than WT cells (Figure 9B). Therefore, our findings revealed that modified LPS from Δ*mliR* strain induced a rougher phenotype in comparison with WT cells. This phenotype was compatible with shorter LPS in Δ*mliR* strain (Figure 9A) as a consequence of a deficit in the synthesis of Kdo-8-P (Figure 8C and Supplementary Figure S5).

## Discussion

**FIGURE 10:**
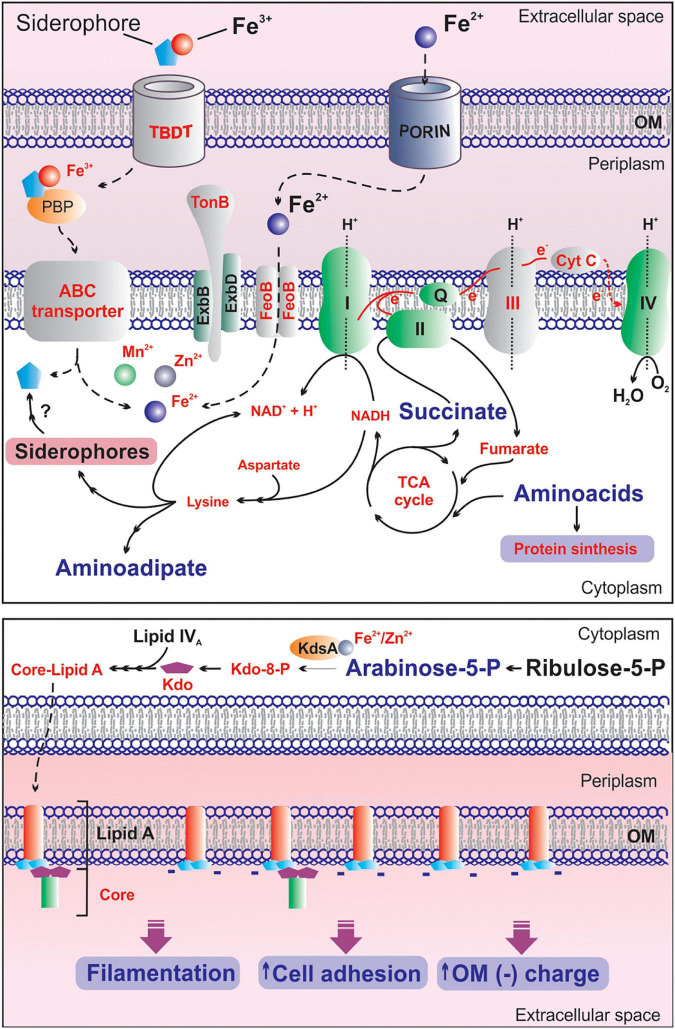
Proposed model on the effects of *mliR* deletion on iron homeostasis and the adaptive responses to intracellular metal deficiency in *B. argentinensis* JUB59. **Upper panel:** the main components of the iron uptake system, the electron transport chain and relevant metabolic pathways are illustrated. Light gray components indicated in red text are downregulated in *mliR*-deletion mutant. Metabolites highlighted in red and small text are decreased in *mliR*-deletion mutant with respect to WT strain. Metabolites highlighted in purple and big text are augmented in *mliR*-deletion mutant with respect to WT strain. See text for more details. **Lower panel:** schematic representation of the biosynthetic pathway of LPS. The lipid component of Lipid A (orange), *N*-acetyl-D-glucosamine phosphate (light blue), KDO, 3-Deoxy-D-manno-octulosonic acid (purple) and the core oligosaccharide (green) are represented. KdsA, Kdo-8-P synthase; OM, outer membrane. See text for more details.

From a biochemical or metabolic perspective, iron is the most valuable metal in biological systems. The central role of this metal makes it a determinant of bacterial pathogenesis, invasiveness and survival (Klebba et al., 2021). A key factor for regulating the intracellular iron availability is the action of various iron uptake systems. In Gram-negative cells TonB-dependent transporters (TBDT) (Morton and Williams, 1989) adsorb ferric iron complexes, and facilitated by TonB-ExbB-ExbD complex, internalize them through the outer membrane (OM) bilayer. Afterward, transport of ferric siderophores across the inner membrane requires a periplasmic binding protein (PBP) and an ABC transporter system. Once the ferric siderophore enters the cytoplasm, ferric ion is reduced to ferrous ion (Fe^2+^), which is destined for storage or incorporation into enzymes (Figure 10). In this context, our findings reveal that deletion of the *mliR* gene in *B. argentinensis* JUB59 causes a significant reduction in the expression of several metal ion transport genes, including two TonB-dependent receptors, a TonB energy transducer, four metal ion ABC transporters and three genes involved in iron uptake. These results are consistent with a significant decrease in the intracellular concentration of iron, manganese and zinc. Therefore, it may be assumed that MliR plays a major role in controlling the expression of ion acquisition genes, crucial to mediate metal homeostasis in cells.

Changes in iron availability require complex control over mechanisms involved in iron uptake. Iron deprivation can lead to death of an organism while an excess of iron can cause damage to cellular machineries through free radical formation via the Fenton Reaction (Fenton, 1894). In bacteria, the ferric uptake regulator (Fur) has been characterized so far as the primary transcription factor controlling components related to iron homeostasis (Chandrangsu et al., 2017). Fur was first described in *E. coli* as an iron-responsive repressor of iron uptake systems and represses its target genes when the intracellular concentration of iron exceeds threshold levels (Fuangthong and Helmann, 2003). In the absence of the metal, Fur-mediated repression is lifted and the iron uptake genes are transcribed (Skaar, 2010). In this context, we here describe a novel transcriptional regulator, not related to the Fur family protein, involved in iron homeostasis in *B. argentinensis* JUB59. Through bioinformatics analyses and structural comparisons we show that MliR is widely conserved in bacteria from a broad range of environments and has remarkable structural differences with members of the MerR and TnrA/GlnR protein families. Furthermore, contrary to Fur, our results suggest that MliR is actually a transcriptional activator devoid of an EBD domain and lacking conserved metal binding sites.

Our findings highlight the importance of MliR in iron homeostasis in *B. argentinensis* JUB59, being essential for sustaining at least 50% of total intracellular iron under non-limiting iron conditions. In this context, given the vital role previously assigned to Fur in iron homeostasis in bacteria, we investigated the presence of Fur homologs in *B. argentinensis* JUB59. By means of sequence homology searches we detected one putative Fur-related gene (*BZARG_RS00045*) in the genome of *B. argentinensis* JUB59. However, further studies are needed to stablish whether *BZARG_RS00045* regulates target genes implicated in iron homeostasis and how it would be related to MliR in terms of iron uptake, utilization and storage systems in *B. argentinensis* JUB59.

In contrast to its ferric counterpart, ferrous iron usually exists in its free form where bacterial transport systems can directly take up the metal. In Gram-negative bacteria, ferrous iron is thought to diffuse freely through the outer membrane porins, so that it can enter the periplasm from where it can be transported into the cytoplasm via different transport systems (Ge and Sun, 2012; Figure 10). The main ferrous iron transport system that is present in both pathogenic and non-pathogenic bacteria is the Feo System (Lau et al., 2015). The *feoB* gene was first discovered in *E. coli* K12 and it encodes a multidomain transmembrane protein that consists of 773 amino acid residues (Kammler et al., 1993; Cartron et al., 2006). *E. coli ΔfeoB* strains are incapable of transporting ferrous iron (Kammler et al., 1993), and many studies have illustrated the importance of FeoB in bacterial virulence (Stojiljkovic et al., 1993; Boyer et al., 2002; Dashper et al., 2005; Aranda et al., 2009; Pandey and Sonti, 2010). Here we show that the mRNA expression levels of a FeoB-associated Cys-rich membrane protein are significantly reduced in Δ*mliR* as compared to WT *B. argentinensis* JUB59. This result suggests that, in addition to TonB-dependent ferric ion uptake, the regulatory role of MliR on iron transport could be extensive to ferrous iron transport systems.

Taken together, these unique features may define MliR as a founding member of a new protein subfamily of transcriptional activators involved in iron homeostasis within the MerR superfamily. Further work needs to be done, however, in order to compare the molecular mechanisms underlying MliR-mediated transcription regulation with respect to cognate members of the MerR and TnrA/GlnR protein families.

In addition to the downregulation of metal ion transport genes, the *mliR*-deletion mutant show significant lower expression of iron utilization genes. Particularly, the expression of complex III and cytochrome C components of the electron transport chain are notably affected in Δ*mliR* mutant. As a consequence, the electron flow through the respiratory chain is expected to be limited in Δ*mliR* cells, affecting the proton-motive force that drives the synthesis of ATP. In this regard, the accumulation of succinate in Δ*mliR* strain accounts for a decrease in the succinate dehydrogenase activity (complex II), which in turn is consistent with a deficiency in the electron transport chain and impacts the functioning of the TCA cycle.

Other genes involved in iron utilization are also downregulated in Δ*mliR* strain: (i) *Mrp/nbp35* (*BZARG_RS14660*), encoding for a putative iron-sulfur (Fe–S) cluster protein that would function as a scaffold to assemble nascent Fe–S clusters for transfer to Fe–S-requiring enzymes (Pardoux et al., 2019) and (ii) *Fe-dependent superoxide dismutase* (*BZARG_RS11940*), encoding an enzyme that catalyzes the de-activation of superoxide and requires iron for activity (Miller, 2012). Additionally, the expression of *BZARG_RS10675* (PepSY domain-containing protein) and *BZARG_RS02985* (OsmC family protein), two genes encoding putative enzymes with antioxidant activity, are as well significantly downregulated in Δ*mliR* strain.

Together, our findings not only provide evidence for the crucial role of MliR in regulating iron uptake systems, but also suggest its involvement in the control of genes related to iron utilization and the enzymatic response to metal-ion-mediated oxidative stress in *B. argentinensis* JUB59. It remains to be determined, however, whether the expression of these genes are directly or indirectly regulated by MliR.

Filamentation allows cells to extend across surfaces, excluding competitors and increasing cell surface area and contact, without substantially decreasing the cell surface-to-volume ratio. An increase in cell surface area enables an enhancement in transport across the membrane when extracellular nutrient concentrations are limiting (Button, 1991; Karasz et al., 2022). In this regard, the filament morphology of Δ*mliR* cell populations seems to arise in response to the deficit in intracellular metal ions concentration to adapt the cell surface area accordingly.

Our results clearly show that Δ*mliR* cells have a low production of Kdo-8-P together with an accumulation of D-arabinose-5-phosphate when compared to WT cells. These findings points toward the idea that the metal-dependent enzyme KdsA could be inhibited in Δ*mliR B. argentinensis* JUB59. In this regard, it has been shown that KdsA activity is strictly dependent on divalent metal ions (Duewel and Woodard, 2000). Therefore, diminished intracellular concentration of iron, zinc and manganese in Δ*mliR* cells could be affecting KdsA activity and, consequently, the synthesis of wild-type LPS. In line with this notion, missense mutations of *kdsA* gene have been linked to instability of the outer membrane, defects in FtsZ-ring formation and filamentous morphology in *E. coli* (Fujishima et al., 2002). In addition, several studies have addressed the relevance on LPS structure in modulating OM charge and facilitating cell attachment to surfaces, biofilm formation and metal uptake (Raetz et al., 2007; Clifton et al., 2016; Clark et al., 2021).

The findings presented here allow us to propose a model on the effects of *mliR* deletion on iron homeostasis and the adaptive response to intracellular metal deficiency in *B. argentinensis* JUB59 (Figure 10). Our results notably integrates transcriptional regulation, metal biology, energy metabolism, filamentation, cell membrane remodeling and cell adhesion, contributing to the understanding of the physiological orchestra that take place in bacteria in response to iron availability.

## Materials and methods

### Bacterial strains and media

Bacterial strains used in this study are listed in Supplementary Table S1. Wild-type and Δ*mliR Bizionia argentinensis* JUB59 strains were grown at 22°C in marine broth 2216 (Difco). Marine conjugation medium was prepared as previously reported (Zhu et al., 2017).

To test the role of iron in *B. argentinensis* JUB59 gene expression, cells were incubated in a modified iron-free Marine Broth 2216 lacking ferric citrate. The water used in media preparation was pre-treated with a Chelex 100 resin (Domingue et al., 1990). Iron-free Marine Broth was supplemented with various concentrations of ferric citrate, as indicated. *Escherichia coli* S17-1 λ pir was cultured in Luria broth (LB) liquid medium or on LB agar at 37°C. When necessary, antibiotics (Sigma) were added at the following concentrations: ampicillin, 100 μg/ml, erythromycin, 4 μg/ml and kanamycin, 50 μg/ml.

### Alignment of protein sequences

Homologous sequences were obtained with Blast^1^ and aligned with the MUSCLE server at the EBI (Madeira et al., 2019). Phylogeny was inferred using the Maximum likelihood method and JTT matrix-based model (Jones et al., 1992). Evolutionary analyses were conducted in MEGA X (Kumar et al., 2018) and tree visualization was performed with Dendroscope 3 (Huson and Scornavacca, 2012).

### Construction of the *mliR* deletion mutant in Wild-type *Bizionia argentinensis* JUB59

Plasmids and primers used in this study are listed in Supplementary Table S1. Our gene deletion strategy was based on a sacB system previously developed in *Z. galactanivorans* (Zhu et al., 2017, Supplementary Figure S2). First, a 2006 bp fragment including the first 18 bp of *mliR* and 1988 bp of upstream sequence was cloned into pYT313 (Zhu et al., 2017) to generate pYT313-1F. Subsequently, a 2036 bp fragment including the last 24 bp of *mliR* and 2012 bp of downstream sequence was cloned into pYT313-1F to generate the deletion construct pYT313-1F-2F. pYT313-1F-2F was introduced into *E. coli* S17-1 λ pir cells by electroporation. *E. coli* S17-1 λ pir cells harboring pYT313-1F-2F were grown overnight in LB supplemented with ampicillin, washed twice with Marine conjugation medium and conjugated with Wild-type *B. argentinensis* JUB59. Afterward, cells were diluted in marine broth and plated on marine agar with erythromycin and kanamycin, to select *B. argentinensis* JUB59 colonies that contained the integrated plasmid after homologous recombination. The erythromycin resistant colonies were inoculated into marine broth medium without antibiotics and incubated with shaking overnight at 22°C to allow for loss of the plasmid by a second recombination event. The *mliR* deletion mutants were selected by growth on marine agar containing 10% sucrose and confirmed by PCR amplification using primers CHF and CHR, which resulted in a 480 bp product from wild-type cells but produced a 170 bp product from cells of the deletion mutant.

### RNA-seq

Wild-type or Δ*mliR B. argentinensis* JUB59 strains were cultured in marine broth at 22°C until the optical density at 600 nm (OD600) reached 1.4. The cells were then centrifuged at 3500 × *g* for 10 min at room temperature. Pellets were washed once with marine broth and total RNA was isolated using Trizol Max Bacterial RNA Isolation Kit (Ambion – Life Technologies) and then treated with TURBO DNAse (Ambion – Life Technologies). RNA samples corresponding to three biological replicates from the wild-type and the *mliR* deletion mutant were submitted to NEB Microbe rRNA depletion kit, TruSeq stranded mRNA library preparation and 100-bp paired-end Illumina NovaSeq6000 sequencing. Removal of Illumina adaptor sequences and low-quality bases at the ends of reads was carried out using Trimmomatic software v0.36 (Bolger et al., 2014). FastQC^2^ was used to assess quality of the reads before and after trimming. Subsequently, reads were aligned to the contigs of the *Bizionia argentinensis* JUB59 genome sequencing project (RefSeq accession number NZ_AFXZ00000000.1) using SAMtools and the Burrows-Wheeler Alignment software (BWA) (Li et al., 2009; Li and Durbin, 2010). Read counts were quantified with the software FeatureCounts (Liao et al., 2014) using the strand specific mode. Differential expression analysis was performed using the DESeq2 software (Love et al., 2014).

### Reverse transcription quantitative real-time PCR

Total wild-type or Δ*mliR B. argentinensis* JUB59 RNA from three independent cultures was isolated as described above. Subsequently, reverse transcription was performed using the first−strand SuperScript III cDNA kit (Invitrogen) and random decamer experiments primers (Invitrogen) in the presence of RNasin ribonuclease inhibitor (Promega). Transcript levels were measured in a Mx3000P Real Time PCR System cycler (Agilent Technologies, Santa Clara, CA, United States) using cDNAs as template, FastStart Universal SYBR Green Master (ROX) (Roche), and the RT-qPCR primers listed in Supplementary Table S1. Transcript levels were normalized to that of the serine hydroxymethyltransferase A (*glyA*) housekeeping as previously reported for members of the Flavobacteriaceae family (Thomas et al., 2011a).

### Biofilm assay

Overnight cultures of wild-type or Δ*mliR B. argentinensis* JUB59 strains were diluted 100-fold in marine broth to an OD600 of 0.05. Then, 24-well plates were inoculated with 1 ml of the diluted culture and incubated for 96 h at 22°C with continuous shaking at 200 rpm. Cultures were removed and washed three times with 1.5 ml of PBS buffer. Next, 1 ml of crystal violet was added, followed by incubation for 15 min at room temperature in darkness. Plates were washed three times with PBS and 1 ml of ethanol was added to each well to remove the attached biomass. Finally, absorbance at 575 nm (OD575) was determined. Blank values (marine broth medium alone) were subtracted from each well value.

### Flow cytometry

Flow cytometry analysis and sorting was carried out on the BD FACSAria Fusion Flow Cytometer, at the Cytometry Facility at the Leloir Institute, Buenos Aires, Argentina. Cells were pelleted and resuspended in PBS (filtered through a 0.2 mm filter) to an OD600 of 0.2–0.3 before analysis and sorting. Cultures were analyzed at 15000–25000 events per second with the purity mask. Signals for FSC and side scatter SSC, area (A), height (H), and width (W) were recorded. Mixed populations encompassing a range of cell lengths were isolated using flow cytometry. Sorting gates were defined based on increasing SSC-W signal. Events in each gate were sorted using a yield mask (130000 events collected per gate). Sorted populations were analyzed via phase-contrast microscopy as described above.

### Microscopy and image analysis

Wild-type and Δ*mliR* cells were harvested by centrifugation (10 min at 8,000 rpm), washed with PBS and diluted 1:100. Aliquots of the suspension (0.1 ml) were stained with 1X CellBrite™ Fix Membrane Dye or Calcofluor White Stain (Biotium). The cells were fixed with 3.7% formaldehyde for 15 min at room temperature with gentle shaking, washed 3 times with PBS and resuspended in PBS to a final OD600 of ∼0.3. The cells incubated with CellBrite™ Fix Membrane Dye were then stained with 250 ng/ml of DAPI (4′, 6′-diamidino-2-phenylindole). The stained cells were immobilized on agarose pads, and visualized by fluorescence microscopy as described below.

Images were captured using a confocal microscope (Carl Zeiss LSM 880) with an objective C Plan-Apochromat 63x/1.4 Oil DIC M27 and Airyscan detector in superresolution mode. For CellBrite stained cells a 543 nm laser and BP 420-480 nm plus BP 495–620 nm filter set were used. Images from DAPI and Calcofluor White stained cells were acquired using excitation laser at 405 nm and BP 420–480 nm plus BP 495–620 nm filter set. Airyscan images were processed and analyzed with the ZEN 2.3 SP1 FP3 Black software. Image sizes were 32,89 × 32,89 μm (932 × 932 pixels).

### Nuclear magnetic resonance – Based metabolomics

Wild-type or Δ*mliR B. argentinensis* (JUB59) strains were cultured in Marine Broth at 22°C until the OD600 reached 1.4. The cells were then centrifuged at 3,500 × *g* for 10 min at 4°C. Cell pellets were washed twice with PBS, resuspended in 2 ml ice-cold 80% methanol, disrupted by sonication and centrifuged at 4°C for 10 min at 15,000 × *g*. Supernatants were collected and dried in a Savant SpeedVac (Thermo Scientific). Dried samples were solubilized in 0.5 ml sodium phosphate buffer (100 mM dissolved in D_2_O, pH = 7.4), supplemented with 3-trimethylsilyl-[2, 2, 3,3,-2H_4_]-propionate (TSP, final concentration 0.33 mM) as chemical shift reference. All NMR experiments were performed at 298 K on a Bruker Avance III spectrometer operating at a proton frequency of 600, 2 MHz and processed as previously reported (Pena et al., 2020). The assignment was achieved using the freely available electronic databases HMDB and BMRB (Ulrich et al., 2008; Wishart et al., 2009, 2013), and subsequently confirmed by 2D spectra including heteronuclear single quantum coherence (HSQC) and total correlation spectroscopy (TOCSY) (Supplementary Table S3).

The NMR spectral areas (AUC) of assigned metabolites were normalized by PQN (Probabilistic Quotient Normalization), subjected to Pareto scaling and analyzed by multivariate analysis using MetaboAnalyst 5.0 (Chong et al., 2018). The statistical significance was assessed by Student’s *t*-test, taking *p* < 0.05 as significant according to false discovery rate.

### Inductively coupled plasma mass spectrometry

WT or Δ*mliR B. argentinensis* JUB59 cells were centrifuged at 3,500 × *g* for 10 min at 4°C. Cell pellets were washed twice with chelated-PBS, resuspended in 15 ml of 2 mM chelated-NTA (nitrilotriacetic acid) in cheated-PBS and incubated for 10 min at 22°C. The cells were then centrifuged at 3,500 × *g* for 10 min at 4°C and washed twice with chelated-PBS. Finally, cells were disrupted by sonication and centrifuged at 4°C for 10 min at 15,000 × *g*. Supernatants were collected and dried in a Savant SpeedVac (Thermo Scientific). A total of six biological replicates of WT and Δ*mliR* dried samples were subjected to metal determination and quantification on an ICP MS (Perkin-Elmer SCIEX, ELAN DRC-e Thornhill, ON, Canada) at the INQUISAL facility (CONICET-UNSL), San Luis, Argentina. Supplementary Table S2 shows the operating conditions of the ICP-MS. Chelex 100 resin (BioRad) was used to deplete solutions of their metal components.

### Lipopolysaccharide analysis

The outer membrane fraction (OMF) of Wild-type and Δ*mliR Bizionia argentinensis* JUB59 was prepared according to Rahman et al. (2002) with minor modifications. Briefly, harvested cells were washed twice with PBS and once with 20 mM Tris–HCl (pH 7,5) by centrifugation (10,000 × *g* for 15 min). After the last wash, the cells were resuspended in 20 mM Tris–HCl and disrupted by sonication on ice for 15 min at 40 W with intervals of 20 s. Whole cell lysate was mixed with 2% Sarkosyl in Tris–HCl for solubilization of the inner membrane and incubated at 25°C for 30 min. The suspension was centrifuged at 10,000 × *g* for 10 min to remove intact cells and the supernatant was then centrifuged at 45,000 × *g* for 1 h at 4°C. The pellet, consisting of OMF, was resuspended in Laemmli sample buffer at 1 mg (dry weight) ml^–1^, and analyzed by SDS-PAGE (15%) in Tris-glycine running buffer and visualized by carbohydrate-specific periodate oxidation and silver staining as previously described (Tsai and Frasch, 1982).

## Importance

The central role of iron in bacteria makes it a determinant of pathogenesis, invasiveness and survival. Significant progress has been made toward the regulation of iron metabolism in model organisms. However, mechanisms of iron homeostasis in Bacteroidetes, one of the dominant phyla in animal gut, soil and oceans, remain largely unknown. Here, we identified a novel transcriptional regulator of the MerR superfamily (MliR), phylogenetically unrelated to cognate Fur proteins, involved in iron homeostasis in the marine bacterium *B. argentinensis* JUB59 and widely conserved in bacteria from a variety of environments. Our results provide evidence for the crucial role of MliR in regulating iron uptake, iron utilization and the enzymatic response to metal-ion-mediated oxidative stress. The findings presented here bring together transcriptional regulation, energy metabolism, membrane remodeling and cell adhesion, providing insights into the global response to iron availability that may raise interest in microbiology in general.

## Data availability statement

The datasets presented in this study can be found in online repositories. The names of the repository/repositories and accession number(s) can be found below: NCBI’s Gene Expression Omnibus (Edgar et al., 2002) and are accessible through GEO Series accession number GSE189140.

## Author contributions

LP: conceptualization, formal analysis, funding acquisition, investigation, methodology, validation, and writing – original draft and review and editing. MB: conceptualization, formal analysis, investigation, methodology, validation, and writing – original draft and review and editing. RS: data curation, formal analysis, validation, and writing – review and editing. MA: conceptualization, formal analysis, funding acquisition, investigation, methodology, validation, writing – original draft and review and editing, project administration, and supervision. LP and MB: collaborated on experiments, analyzed the data, and contributed to the writing of the manuscript in a complementary and essential way. All authors contributed to the article and approved the submitted version.

## Footnotes

1
https://blast.ncbi.nlm.nih.gov/Blast.cgi

2
www.bioinformatics.babraham.ac.uk/projects/fastqc/

## Supplementary material

Supplementary Figure S1 AlphaFold-modeled structure of the MliR protein of *Bizionia argentinensis* JUB59. Relevant N-terminal residues are labeled and highlighted in green.

Supplementary Figure S2 Site-directed mutagenesis of *B. argentinensis* JUB59. **(A)** The genomic context of the *mliR* gene and the primers used to identify sucrose-resistant deletion mutants are displayed. **(B)** Colony PCR of 9 selected sucrose-resistant colonies (as described under section “Materials and methods”). WT, wild type strain of *B. argentinensis* JUB59; M: PCR product from primers 3 and 4; Primer 1: CHF (Supplementary Table S1); Primer 2: CHR (Supplementary Table S1).

Supplementary Figure S3 Merged fluorescence and phase contrast images of DAPI-stained Δ*mliR B. argentinensis* JUB59 cells grown into exponential phase. The yellow arrows highlight division events.

Supplementary Figure S4 PLS-DA score plot derived from the 600 MHz ^1^H NMR spectra of WT and Δ*mliR B. argentinensis* JUB59 cells. The predictive ability of the PLS-DA model was calculated via cross-validation (CV) (R2: 0.999, Q2: 0.986).

Supplementary Figure S5 Box plot of scaled ^1^H-NMR AUC (area under the curve) of metabolites identified in Δ*mliR* and WT *B. argentinensis* JUB59 strains. In the light brown panel and the violet panel, statistically significant and no significant varying metabolites are grouped, respectively.

Supplementary Figure S6 Enrichment analysis of significant differentially expressed genes in Δ*mliR* strain. The enrichment analysis was performed with genes that displayed log_2_-fold change > 1 and presented annotated data on its molecular function, biological process or sub-cellular localization at the Uniprot database. The log_2_-fold change of downregulated (blue) and upregulated (red) genes in Δ*mliR* strain are plotted. The proportions of significant differentially expressed genes found in different biological processes are shown in pie charts.

Supplementary Table S1 Bacterial strains, vectors and primers.

Supplementary Table S2 ICP-MS conditions for metal analysis.

Supplementary Table S3 Global transcriptional analysis of WT and Δ*mliR* strains of *B. argentinensis* JUB59. The genes with statistically significant differential expression between Δ*mliR* and WT cells (log_2_-fold change > 1 and *P*-value < 0.05) are listed below.

Supplementary Table S4 ^1^H-NMR assignments of 32 metabolites from *B. argentinensis* JUB59.

Supplementary Table S5 Significantly varying metabolites between WT and Δ*mliR* strains of *B. argentinensis* JUB59. The statistics from Student’s *t*-test are shown. FDR, false discovery rate.

Supplementary Table S6 Sequences and accession numbers of MliR homologous proteins included in the phylogenetic analysis of Figure 1C.
