# Supplementary figures and images for: MliR, a novel MerR-like regulator of iron homeostasis, impacts metabolism, membrane remodeling, and cell adhesion in the marine Bacteroidetes Bizionia argentinensis
Source: Front Microbiol. 2022 Sep 2;13:987756. doi: 10.3389/fmicb.2022.987756 (PMC9478572; doi:10.3389/fmicb.2022.987756)

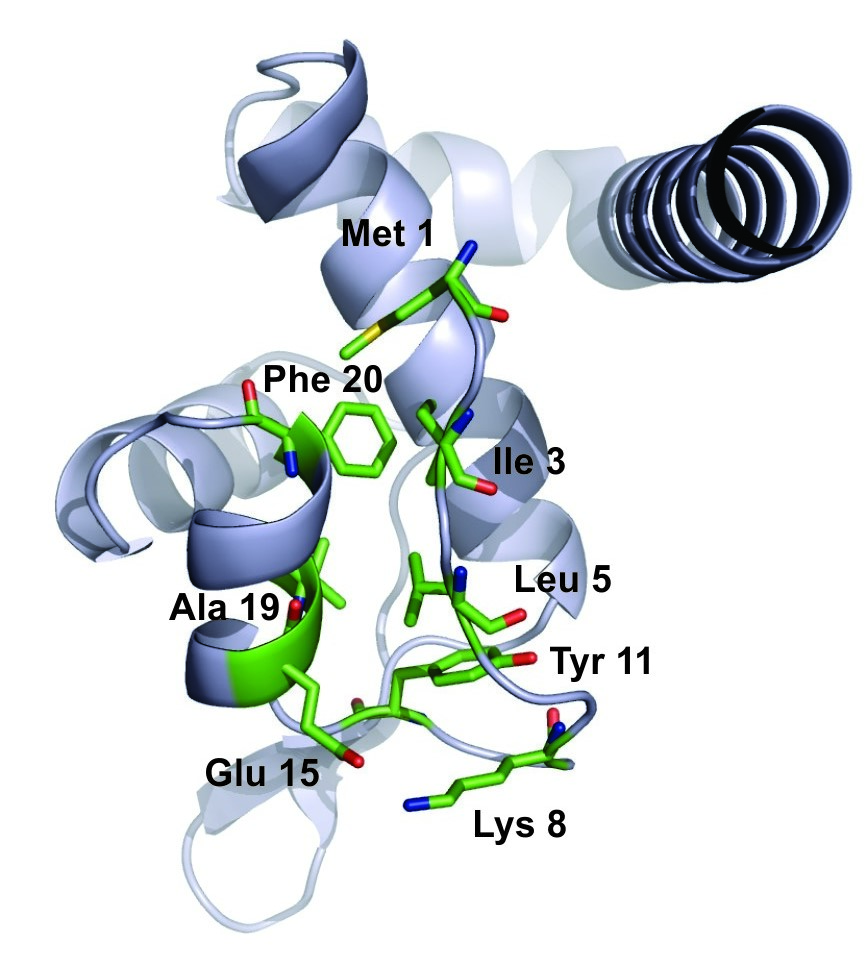

Supplement: Supplementary Figure S1 — AlphaFold-modeled structure of the MliR protein of Bizionia argentinensis JUB59. Relevant N-terminal residues are labeled and highlighted in green. [file Image_1.JPEG]

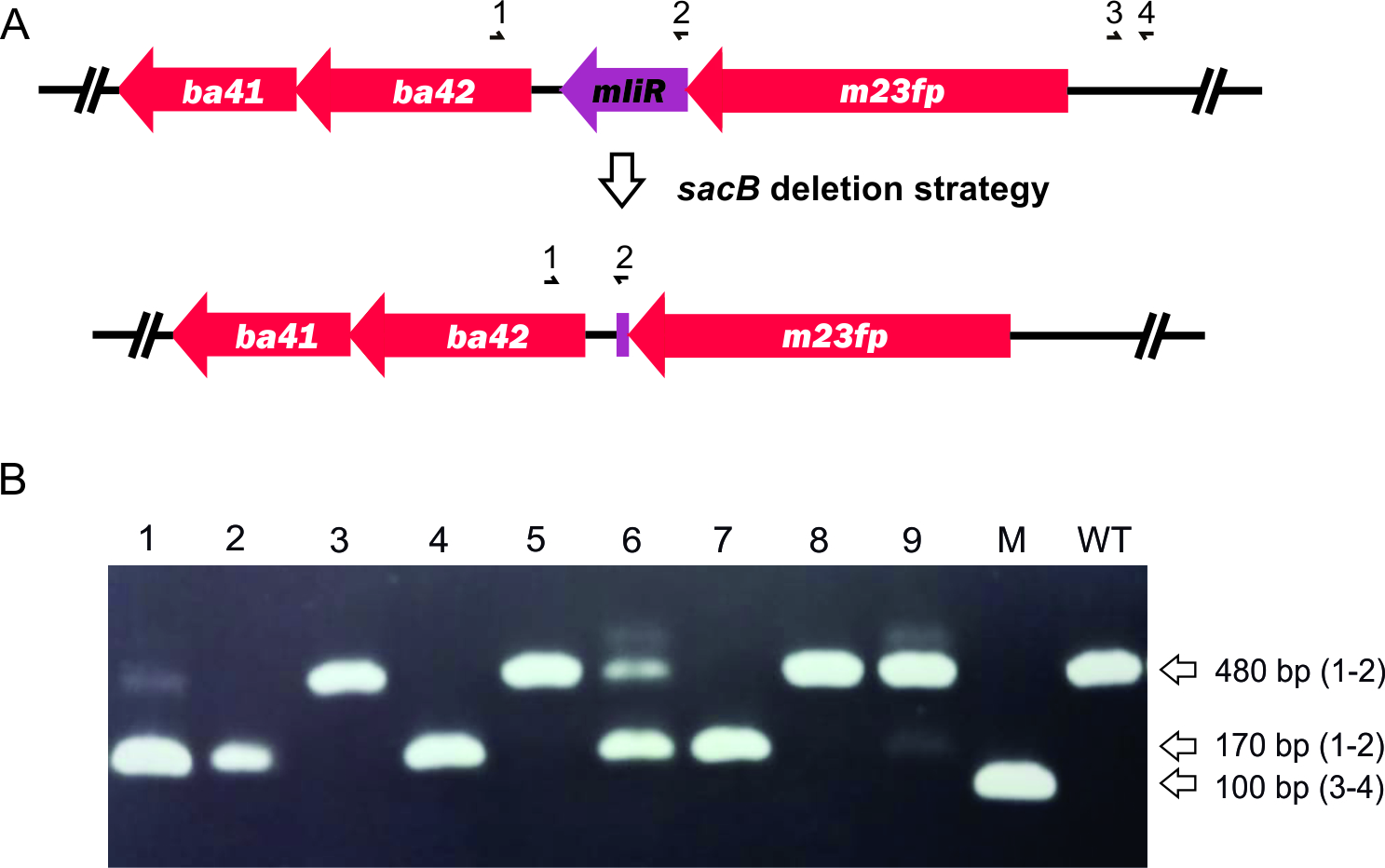

Supplement: Supplementary Figure S2 — Site-directed mutagenesis of B. argentinensis JUB59. (A) The genomic context of the mliR gene and the primers used to identify sucrose-resistant deletion mutants are displayed. (B) Colony PCR of 9 selected sucrose-resistant colonies (as described under section “Materials and methods”). WT, wild type strain of B. argentinensis JUB59; M: PCR product from primers 3 and 4; Primer 1: CHF (Supplementary Table S1); Primer 2: CHR (Supplementary Table S1). [file Image_2.JPEG]

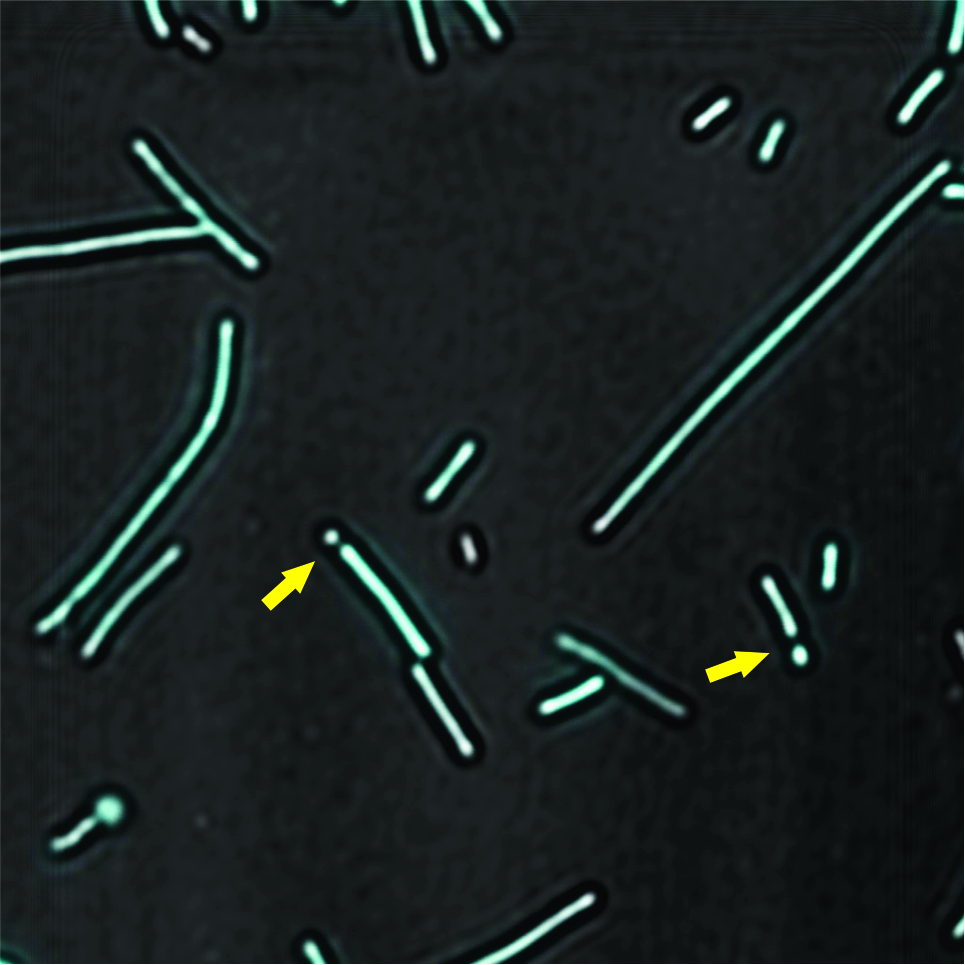

Supplement: Supplementary Figure S3 — Merged fluorescence and phase contrast images of DAPI-stained ΔmliR B. argentinensis JUB59 cells grown into exponential phase. The yellow arrows highlight division events. [file Image_3.JPEG]

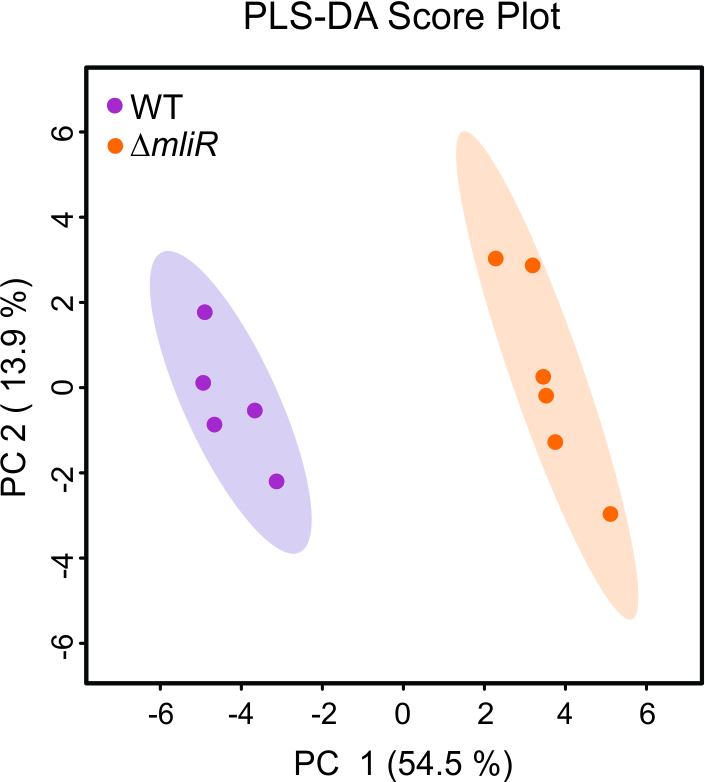

Supplement: Supplementary Figure S4 — PLS-DA score plot derived from the 600 MHz 1H NMR spectra of WT and ΔmliR B. argentinensis JUB59 cells. The predictive ability of the PLS-DA model was calculated via cross-validation (CV) (R2: 0.999, Q2: 0.986). [file Image_4.JPEG]

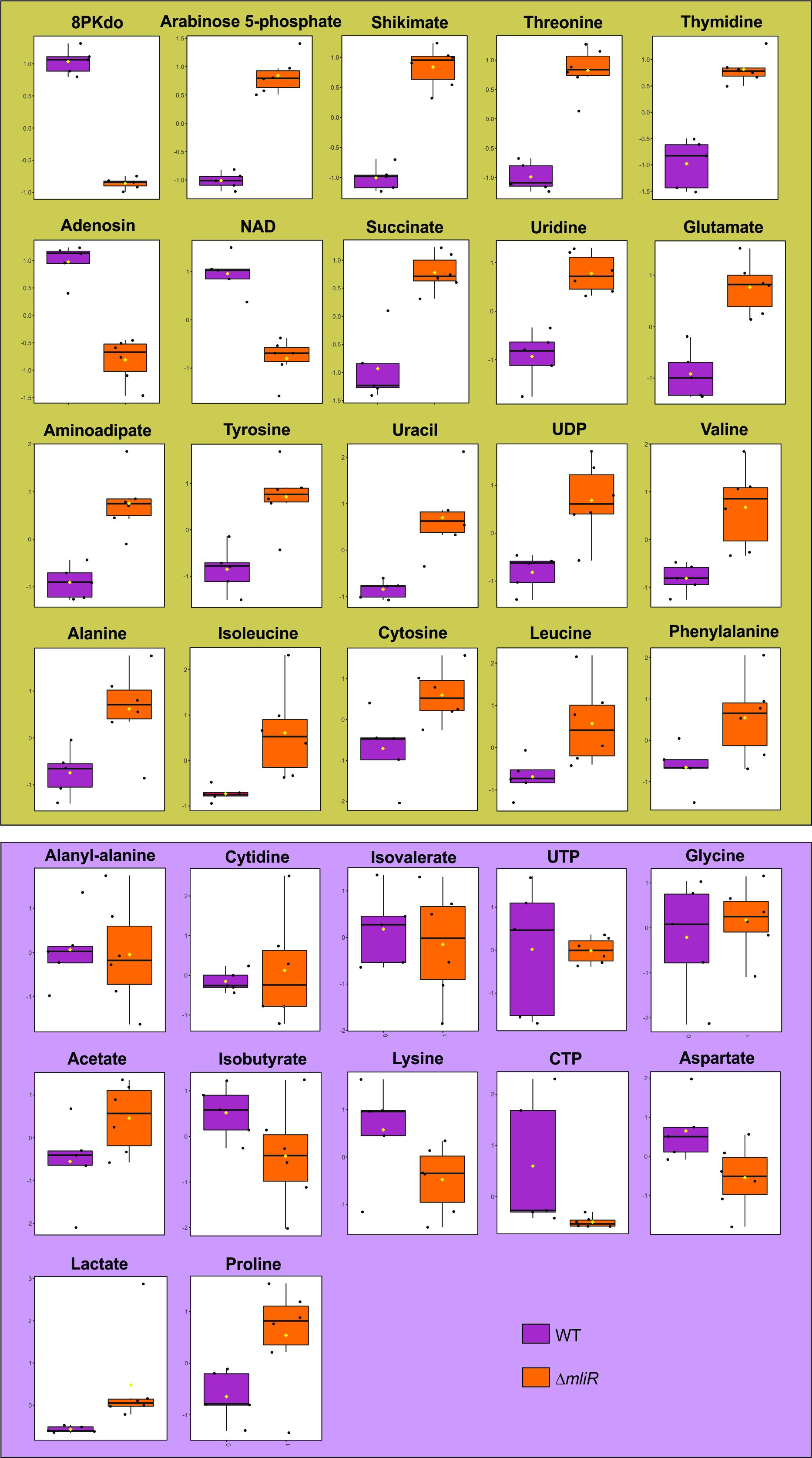

Supplement: Supplementary Figure S5 — Box plot of scaled 1H-NMR AUC (area under the curve) of metabolites identified in ΔmliR and WT B. argentinensis JUB59 strains. In the light brown panel and the violet panel, statistically significant and no significant varying metabolites are grouped, respectively. [file Image_5.JPEG]

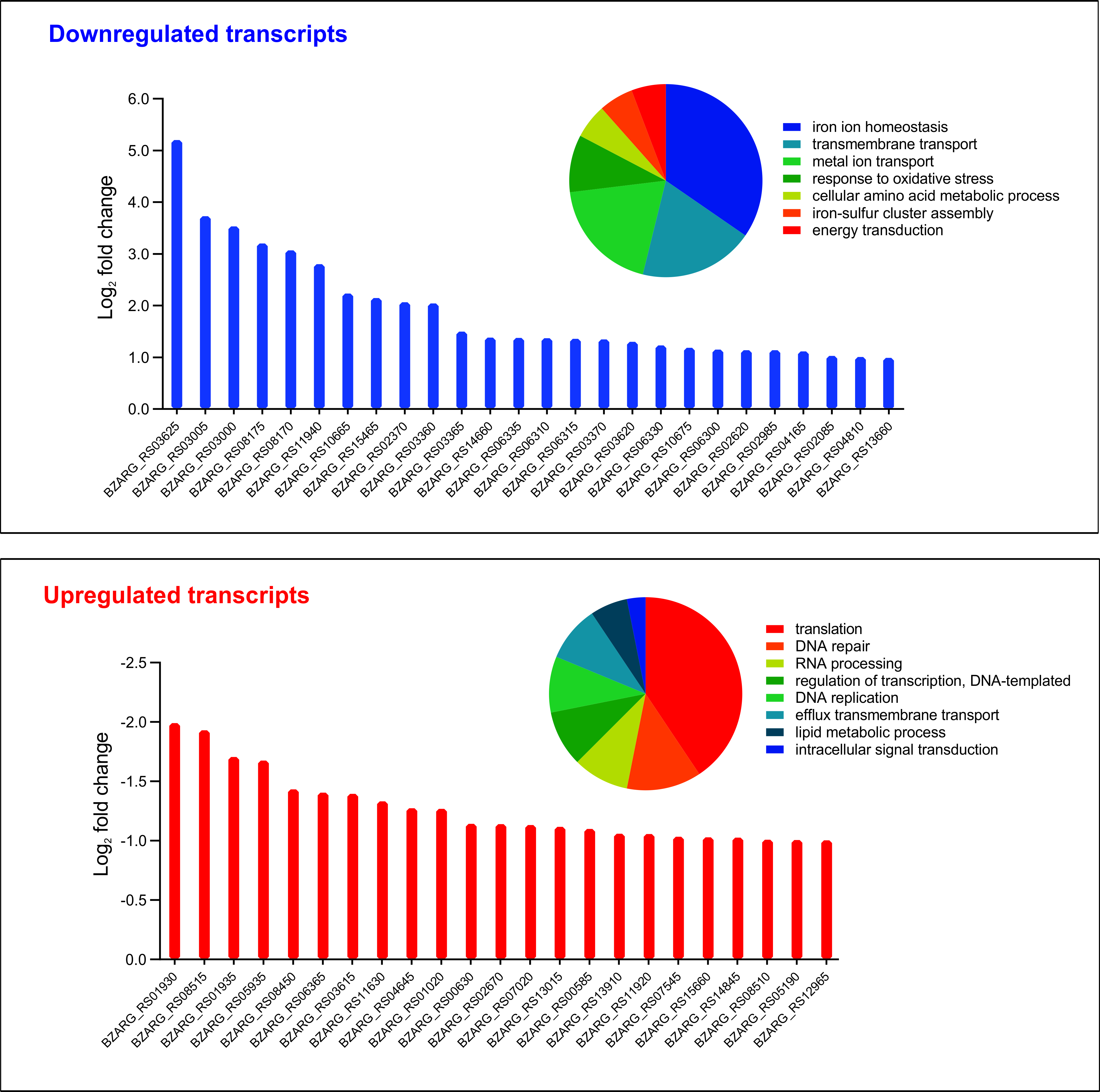

Supplement: Supplementary Figure S6 — Enrichment analysis of significant differentially expressed genes in ΔmliR strain. The enrichment analysis was performed with genes that displayed log2-fold change > 1 and presented annotated data on its molecular function, biological process or sub-cellular localization at the Uniprot database. The log2-fold change of downregulated (blue) and upregulated (red) genes in ΔmliR strain are plotted. The proportions of significant differentially expressed genes found in different biological processes are shown in pie charts. [file Image_6.JPEG]
