# Supplementary material for: MliR, a novel MerR-like regulator of iron homeostasis, impacts metabolism, membrane remodeling, and cell adhesion in the marine Bacteroidetes Bizionia argentinensis
Source: Front Microbiol. 2022 Sep 2;13:987756. doi: 10.3389/fmicb.2022.987756 (PMC9478572; doi:10.3389/fmicb.2022.987756)
Supplement: Supplementary Table S1 — Bacterial strains, vectors and primers. [file Table_1.pdf]

ICP MS conditions

RF Forward power	1050 W
**Gas flow rates:**	
Plasma	13 L. min^{-1}
Auxiliary	1.35 L.min^{-1}
Nebulizer	0.75 L.min^{-1}
Resolution	Normal
Scanning mode	Peak hop
Dwell time	500 ms
