# Supplementary material for: MliR, a novel MerR-like regulator of iron homeostasis, impacts metabolism, membrane remodeling, and cell adhesion in the marine Bacteroidetes Bizionia argentinensis
Source: Front Microbiol. 2022 Sep 2;13:987756. doi: 10.3389/fmicb.2022.987756 (PMC9478572; doi:10.3389/fmicb.2022.987756)
Supplement: Supplementary Table S2 — ICP-MS conditions for metal analysis. [file Table_2.pdf]

Downregulated

Gene	*Name*	*Molecular function*	*log_2-fold change*
BZARG_RS03625	TonB-dependent receptor	Siderophore uptake transmembrane transporter activity	5.2
BZARG_RS03005	Rieske (2Fe-2S) protein	Iron-sulfur cluster binding	3.7
BZARG_RS03000	Cytochrome c	Electron transfer activity	3.5
BZARG_RS08175	D-serine ammonia-lyase	D-serine ammonia-lyase activity	3.2
BZARG_RS08170	AraC binding Domain	Sustrate binding activity	3.1
BZARG_RS11940	Superoxide dismutase	Superoxide dismutase activity	2.8
BZARG_RS10665	Ankyrin repeat domain-containing protein	Unknown function	2.2
BZARG_RS15465	FeoB associated Cys rich membrane protein	Unknown function	2.1
BZARG_RS02370	Asparagine synthase B	Asparagine synthetase (glutamine-hydrolyzing) activity	2.1
BZARG_RS03360	Imelysin family protein	Unknown function	2.0
BZARG_RS03365	HTTM domain-containing protein	Unknown function	1.5
BZARG_RS14660	Mrp/NBP35 family protein	Fe-S cluster assembling	1.4
BZARG_RS06335	Fe^{2+} transport protein A	Metal ion binding	1.4
BZARG_RS06310	Mn^{2+}/Zn^{2+} ABC transporter	Metal ion binding	1.4
BZARG_RS06315	Zn^{2+} ABC transporter	Metal ion binding	1.4
BZARG_RS03370	TonB-dependent receptor	Siderophore uptake transmembrane transporter activity	1.3
BZARG_RS03620	AraC transcriptional regulator	DNA binding activity	1.3
BZARG_RS06330	Fe2+ transport protein B	Metal ion binding	1.2
BZARG_RS10675	PepSY domain-containing protein	Oxidoreductase activity	1.2
BZARG_RS06300	Mn^{2+}/Zn^{2+} ABC transporter	Metal ion binding	1.2
BZARG_RS02620	Fe^{3+} ABC transporter	Metal ion binding	1.1
BZARG_RS02985	OsmC family protein	Peroxidase activity	1.1
BZARG_RS04165	Energy transducer TonB	energy transducer activity	1.1
BZARG_RS02085	Rhodanese-like domain-containing protein	Unknown function	1.0
BZARG_RS04810	3-hydroxyanthranilate 3,4-dioxygenase	3-hydroxyanthranilate 3,4-dioxygenase activity	1.0
BZARG_RS13660	Glu/Leu/Phe/Val dehydrogenase	Oxidoreductase activity	1.0

Upregulated

Gene	*Name*	*Molecular function*	*log_2-fold change*
BZARG_RS01930	DNA polymerase IV	DNA polymerase activity	-2.0
BZARG_RS01935	DNA polymerase III subunit alpha	DNA polymerase activity	-1.7
BZARG_RS01830	DoxX family protein	Unknown fuction	-1.5
BZARG_RS11630	T9SS type A sorting domain-containing protein	Unknown fuction	-1.3
BZARG_RS04645	thioredoxin domain-containing protein	Oxidoreductase activity	-1.3

BZARG_RS01020	SEC-C motif family protein	Unknown fuction	-1.3
BZARG_RS00630	GNAT family N-acetyltransferase	Transferase activity	-1.1
BZARG_RS07020	single-stranded DNA-binding protein	DNA binding	-1.1
BZARG_RS13015	DMT family transporter	Transmembrane transporter activity	-1.1
BZARG_RS00585	helix-turn-helix transcriptional regulator	DNA binding	-1.1
BZARG_RS13910	choice-of-anchor D domain-containing protein	Unknown fuction	-1.1
BZARG_RS11920	30S ribosomal protein S21	Structural constituent of ribosome	-1.1
BZARG_RS07545	T9SS type A sorting domain-containing protein	Unknown fuction	-1.0
BZARG_RS15660	T9SS type A sorting domain-containing protein	Unknown fuction	-1.0
BZARG_RS14845	KTSC domain-containing protein	Chaperone activity	-1.0
BZARG_RS08510	GIY-YIG nuclease family protein	Endonuclease activity	-1.0
BZARG_RS05190	DEAD/DEAH box helicase	RNA helicase activity	-1.0

tRNAs

Gene	***Name***	***Molecular function***	***log_2-fold change***
BZARG_RS08515	tRNA-Phe		-1.9
BZARG_RS05935	tRNA-Met		-1.7
BZARG_RS06365	tRNA-Gln		-1.4
BZARG_RS03615	tRNA-Pro		-1.4
BZARG_RS02670	tRNA-Leu		-1.1
BZARG_RS12965	tRNA-Lys		-1.0
