# Supplementary material for: MliR, a novel MerR-like regulator of iron homeostasis, impacts metabolism, membrane remodeling, and cell adhesion in the marine Bacteroidetes Bizionia argentinensis
Source: Front Microbiol. 2022 Sep 2;13:987756. doi: 10.3389/fmicb.2022.987756 (PMC9478572; doi:10.3389/fmicb.2022.987756)
Supplement: Supplementary Table S3 — Global transcriptional analysis of WT and ΔmliR strains of B. argentinensis JUB59. The genes with statistically significant differential expression between ΔmliR and WT cells (log2-fold change > 1 and P-value < 0.05) are listed below. [file Table_3.pdf]

N°	Metabolite	Groups	δ ¹H (ppm)	Multiplicity
1	**Acetate**	CH_3	1,90	(s)
2	**Adenosine**	CH (adenine)	8.14	(s)
		CH' (adenine)	8,30	(s)
3	**Alanine**	β–CH_3	1,47	(d)
4	**Alanyl-alanine**	β–CH_3	1.53	(d)
		β–CH_3´	1.35	(d)
		α–CH	4.07	(tocsy)
		α–CH´	4.14	(tocsy)
5	**2-Aminoadipate**	α–CH	3.73	(tocsy)
		β–CH_2	1.85	(tocsy)
		γ–CH_2	1.63	(tocsy)
		δ–CH_2	2.23	(t)
6	**Arabinose-5-phosphate**	H1 (-OH-CH)	5.23	(d)
		H2 (-OH-CH)	3.94	(tocsy)
		H3 (-OH-CH)	3.94	(tocsy)
		H4 (-OH-CH)	3.31	(tocsy)
		H5 (CH_2)	3.53	(tocsy)
7	**Aspartate**	β–CH_2	2,80	(dd)
		β–CH_2	2.67	(tocsy)
		α–CH	3,89	(tocsy)
8	**Cytidine triphosphate (CTP)**	2-CH (cytosine)	6.12	(tocsy)
		3-CH (cytosine)	8.06	(d)
		1´-CH (ribose)	4.36	(tocsy)
		2´-CH (ribose)	4.47	(tocsy)
		3´-CH (ribose)	4.41	(tocsy)
		4´-CH (ribose)	6.07	(tocsy)
		5´-CH_2 (ribose)	4.36	(tocsy)
9	**Cytidine**	2-CH (cytosine)	5.91	(tocsy)
		3-CH (cytosine)	7.86	(d)
		1´-CH (ribose)	5.9	(tocsy)
		2´-CH (ribose)	4.36	(tocsy)
		3´-CH (ribose)	4.24	(tocsy)
		4´-CH (ribose)	4.14	(tocsy)
10	**Cytosine**	2-CH	5.98	(tocsy)
		3-CH	7.49	(d)
11	**Glutamate**	γ–CH_2	2,34	(m)
		β–CH_2	2,05	(m)
		α–CH	3,75	(dd)
12	**Glycine**	CH_2	3.55	(s)
13	**Isoleucine**	α–CH	3.67	(tocsy)

		β–CH	1.97	(tocsy)
		γ1–CH_2	1.46, 1.25	(tocsy)
		γ2–CH_3	0,99	(d)
		δ–CH_3	0.93	(tocsy)
14	**Isobutyrate**	α–CH	2.37	(tocsy)
		β1–CH_2	1.05	(d)
		β2–CH_3	1.05	(d)
15	**Isovalerate**	β–CH_2	1.93	(tocsy)
		γ–CH	2.03	(tocsy)
		δ–CH_3	0.89	(d)
		δ–CH_3´	0.89	(d)
16	**3-deoxy-manno-octulosonate-8-phosphate (8PKdo)**	3´-CH (ax)-α	1.78	(dd)
		3´-CH (ax)-β	1.82	(dd)
		3´-CH (eq)	2.18	(dd)
		4´-CH	3.93	(tocsy)
		5´-CH	3.79	(m)
		6´-CH	3.43	(t)
		7´-CH	3.56	(tocsy)
		8´-CH_2	4.09	(tocsy)
		8´-CH_2	3.95	(tocsy)
17	**Lactate**	α–CH	4,10	(q)
		β–CH_3	1.32	(d)
18	**Leucine**	α–CH	3.74	(tocsy)
		β–$CH_{2,}$ γ-CH	1.70	(tocsy)
		δ–CH_3	0,95	(t)
		δ'-CH_3	0.95	(t)
19	**Lysine**	α–CH	3.76	(tocsy)
		β–CH_2	1.9	(tocsy)
		γ–CH_2	1.47	(tocsy)
		δ–CH_2	1.72	(tocsy)
		ε–CH_2	3.02	(t)
20	**Nicotinamide adenine dinucleotide, oxidized (NAD^+)**	3-CH (nicotinamide)	9.32	(s)
		4-CH (nicotinamide)	9.15	(d)
		5-CH (nicotinamide)	8.20	(m)
		6-CH (nicotinamide)	8.85	(d)
		2-CH (adenosine)	8.41	(s)
		4-CH (adenosine)	8.16	(m)
21	**Phenyalanine**	δ1–CH	7.32	(d)
		δ2–CH	7.32	(d)
		ε1–CH	7.42	(m)
		ε2–CH	7.42	(m)

		ζ–CH	7.37	(m)
22	**Proline**	α–CH	4.12	(dd)
		β–CH_2	2.33	(tocsy)
		γ–CH_2	2.01	(tocsy)
		δ–CH_2	3.30, 3.40	(tocsy)
23	**Threonine**	α–CH	3.58	(tocsy)
		β–CH	4.23	(tocsy)
		γ–CH_3	1.32	(d)
24	**Thymidine**	CH (thymine)	7.63	(s)
		CH_3 (thymine)	1.89	(tocsy)
		4´-CH (ribose)	6.29	(t)
25	**Tyrosine**	δ1–CH	7.18	(d)
		δ2–CH	7.18	(d)
		ε1–CH	6.89	(d)
		ε2–CH	6.89	(d)
26	**UDP**	2-CH (uracil)	5.96	(tocsy)
		3-CH (uracil)	7.94	(m)
		1´-CH (ribose)	4.23	(tocsy)
		2´-CH (ribose)	4.35	(tocsy)
		3´-CH (ribose)	4.35	(tocsy)
		4´-CH (ribose)	5.94	(tocsy)
		5´-CH_2 (ribose)	4.23	(tocsy)
27	**Uracil**	2-CH	7.53	(d)
		3-CH	5.79	(tocsy)
28	**Uridine**	2-CH (uracil)	7.86	(d)
		3-CH (uracil)	5.89	(tocsy)
29	**UTP**	2-CH (uracil)	5.97	(tocsy)
		3-CH (uracil)	8.09	(d)
		1´-CH (ribose)	4.22	(tocsy)
		2´-CH (ribose)	4.36	(tocsy)
		3´-CH (ribose)	4.36	(tocsy)
		4´-CH (ribose)	5.93	(tocsy)
		5´-CH_2 (ribose)	4.22	(tocsy)
30	**Shikimate**	2-CH	3.69	(tocsy)
		3-CH	3.97	(tocsy)
		4-CH	6.42	(m)
		5-CH	4.39	(tocsy)
		6-CH_2	2.75, 2.18	(tocsy)
31	**Succinate**	2-CH_2	2.39	(s)
		3-CH_2	2.39	(s)
32	**Valine**	α–CH	3.60	(tocsy)

	β–CH	2.27	(tocsy)
	δ–CH_3	1.03	(d)
	δ'-CH_3	0.98	(d)

Note: d, doublet ; m, multiplet ; s, singlet ; t, triplet, tocsy; resonances assigned from ^1H-^1H Tocsy experiment.
