# Supplementary material for: MliR, a novel MerR-like regulator of iron homeostasis, impacts metabolism, membrane remodeling, and cell adhesion in the marine Bacteroidetes Bizionia argentinensis
Source: Front Microbiol. 2022 Sep 2;13:987756. doi: 10.3389/fmicb.2022.987756 (PMC9478572; doi:10.3389/fmicb.2022.987756)
Supplement: Supplementary Table S4 — 1H-NMR assignments of 32 metabolites from B. argentinensis JUB59. [file Table_4.pdf]

Metabolite	t stat	p value	FDR
**3-Deoxy-D-manno-octulosonate 8-phosphate**	21.02	5.87E-09	1.88E-07
**Arabinose 5-phosphate**	-11.76	9.12E-07	1.46E-05
**Shikimate**	-10.57	2.25E-06	2.40E-05
**Threonine**	-8.79	1.04E-05	8.28E-05
**Thymidine**	-7.96	2.30E-05	1.39E-04
**Adenosine**	7.84	2.60E-05	1.39E-04
**NAD**	6.98	6.45E-05	2.95E-04
**Succinate**	-5.96	2.13E-04	7.56E-04
**Uridine**	-5.96	2.13E-04	7.56E-04
**Glutamate**	-5.56	3.52E-04	1.13E-03
**Aminoadipate**	-5.22	5.48E-04	1.59E-03
**Tyrosine**	-4.23	2.19E-03	5.85E-03
**Uracil**	-4.11	2.63E-03	6.47E-03
**UDP**	-3.80	4.22E-03	9.64E-03
**Valine**	-3.70	4.88E-03	1.04E-02
**Alanine**	-3.03	1.43E-02	2.86E-02
**Isoleucine**	-2.94	1.65E-02	3.12E-02
**Cytosine**	-2.78	2.13E-02	3.79E-02
**Leucine**	-2.61	2.85E-02	4.79E-02
**Phenylalanine**	-2.60	2.86E-02	4.81E-02
