# Supplementary material for: MliR, a novel MerR-like regulator of iron homeostasis, impacts metabolism, membrane remodeling, and cell adhesion in the marine Bacteroidetes Bizionia argentinensis
Source: Front Microbiol. 2022 Sep 2;13:987756. doi: 10.3389/fmicb.2022.987756 (PMC9478572; doi:10.3389/fmicb.2022.987756)
Supplement: Supplementary Table S5 — Significantly varying metabolites between WT and ΔmliR strains of B. argentinensis JUB59. The statistics from Student’s t-test are shown. FDR, false discovery rate. [file Table_5.pdf]

Strains	
**Name**	
Bizionia argentinensis JUB59	
E. coli S17-1 λpir	
**Plasmids**	
**Name**	
pYT313	*sacB*-containing suicide vector; Ap^r (Em^r)*
**Primers**	
**Name**	**Sequence**
1FrBamF	GGATCCCCATTTTAGTTTCTGCTAAAATGG
1FrXbaIR	TCTAGACGTCAAAATTAAAGAACAGTTGTAAC
2FrXbaIF	TCTAGACGGGTAAGTTGATATGCATTAGTC
2FrSphIR	GCATGCGTTTCAATTTCACGAATAATTGGC
CHF	AGAATAAAGTCCTATTGCAAT
CHR	CATTTACATTATGAGATATTT
MliR_F	AATGCCAAAGGCAATAGAAAGT
MliR_R	GCGCCTTCTAAAGTGAAACC
BZARG_RS03370_F	TACGGTTGCTGCTGCTTATAG
BZARG_RS03370_R	AGGACCACCTTCTTGTTGTG
BZARG_RS03625_F	CGCTTAGATGGTGCTGGTAA
BZARG_RS03625_R	AACTCAAGCCTAAACCCAGTAG
BZARG_RS03005_F	CTTAATGTAGGCGGCGGAATA
BZARG_RS03005_R	GATCGGCTGGTCCTTCTAATAC
BZARG_RS06310_F	TGCCGTTCAAGTAGACGATTTA
BZARG_RS06310_R	CAGCACCATTAGGACCAACA
BZARG_RS06315_F	CAAGCCGCTGTCCAATCTAA
BZARG_RS06315_R	TTCTACCGTTCCCGCATTTC
BZARG_RS01935_F	CGGGCGCGTATTATGTAGAA
BZARG_RS01935_R	CCTGAAACGCCTGGTCTAAT
BZARG_RS01930_F	TCCGGAAGCAGTGGTTATTC
BZARG_RS01930_R	CTGGCACACGCTCCTTAATA
BZARG_RS11940_F	GGCTAGCACAGGACAATGAA
BZARG_RS11940_R	TCCCAAACGTCTAGGGTTAGTA
BZARG_RS08170_F	GGAGATTTAATTGCGTTGGAAGG
BZARG_RS08170_R	CCCGCTTTGCAGAGTCTAAT
BZARG_RS08175_F	GCTTTACAACCTGCTCACTAAAC
BZARG_RS08175_R	GAACCATTTGCGGACCAATAAG
BZARG_RS03000_F	GTCACGCTGGAGATGGTAAA
BZARG_RS03000_R	GTGGGCCACCTTGATCTAAATA
BZARG_RS11415_F (*glyA*)	ATGGAACGCGACGAACAG
BZARG_RS11415_R (*glyA*)	CCATGACCTGGTCACTAACAAAG
Primer 3	TGTTTTTGTGACTTACTAATACTC
Primer 4	TTTGTAAGCTTATGTTAAAATTC

*Erythromycin resistance is expressed in *B. argentinensis* JUB59
