# Supplementary material for: MliR, a novel MerR-like regulator of iron homeostasis, impacts metabolism, membrane remodeling, and cell adhesion in the marine Bacteroidetes Bizionia argentinensis
Source: Front Microbiol. 2022 Sep 2;13:987756. doi: 10.3389/fmicb.2022.987756 (PMC9478572; doi:10.3389/fmicb.2022.987756)
Supplement: Supplementary Table S6 — Sequences and accession numbers of MliR homologous proteins included in the phylogenetic analysis of Figure 1C. [file Data_Sheet_6.PDF]

>WP_008634818.1 - *Bizionia argentinensis*
MHINLPEKRYYGIGEVAKAFGVNASLIRFWEKEFDVLQPKKNAKGNRKFTPEDIQNLKLIYHLVKERGFT
LEGAKTHLKEGKQETLNMFQIISKLKEVKAKLVKIKEQL

>ACS34466.1 - *Thermococcus gammatolerans*
MRIINAEGLILGRLASKVAKMLLEGEEVVIVNAEKAIITGNREDIFAKYKQRTELRTRTNPRRGPFYPKR
SDEIVRRTVRGMLPWKTDRGRKAFKRLKVYVGVPKEFEGKELETISEAHMSRLATPKYVTVGEVAKFLGG
KF

>PZR34194.1 - *Azospira oryzae*
MAYKEKEIEKIYYSIGEVAEMFDVAPSLIRFWESEFELIQPKKNRKGNRQFTKEDIDNVRTIYHLVKEKG
FTLQGAKEMLKNDTQSIKDKMGLLESLKKVRSFLVEVREKLH

>WP_182318972.1 - *Wolbachia pipientis*
MNKEKLFYTIGEVAEELHLEQHVLRFWEGQFHQIKPTKRKGRRLYDRKCIEAIKKVKYMLYDKGYTIKGV
QKEFGNDIKVTKNLLQELTDLRDYLTSKINNEESNKQT

>MBC7606425.1 - *Burkholderiales* bacterium
MHIELSPEKRYFSIGELARAFGVNASLIRFWDKEFDILKPKKNAKGNRMFTPEDVKNLQLIYHLVKERGF
TLEGAKTHLKEGQKKTLDKFEIISKLEGIKVQLQHIKNEL

>PHO01602.1 - *Rhodobacteraceae* bacterium
MYVDLPDKRYYKIGEVAKAFGVNSSLIRFWDKEFEIINPKKNAKGDRLFTQEDVKNFKIIYNLVKERGFT
LDGAKQKLKKNPEGVLNNQEIISRLETVKAELIKIKNQL

>WP_024071740.1 - *Ehrlichia muris*
MNEDKLLQSEYSGKLKTIGEVAKDLNVEQYVLRFWEEKFPQINPIKRRGRRLYSQVDIDTLKYIKYLLYD
KGYKIKGVQQELNKKTAKVSKNNNNMDHSIYKESLGRVLDEILNLRDNLLEKLNGM

>WP_011964047.1 - *Flavobacterium psychrophilum*
MHIDLPKDKRYYSMGEIAKAFEVNQSLIRFWDKEFDILKPKKNAKGNRMFTPEDVQHLQLIFHLVKERGF
TLEGAKVRLKEGQKKTLDKFAIVSKLESIRSQLINIKNEL

>MBR9914672.1 - *Algicola sp.*
MHIDLPEKRYYGIGEVAKAFDVNTSLIRFWEKEFDILKPKKNAKGNRKFTPEDIKNLKFIYHLVKERGFT
LEGAKTHLKEEKKESLDKFQIIDKLEGIKAQLIKIKSQL

>WP_069606326.1 - *Leptospira tipperaryensis*
MKKKPNPAKDEDSNQKELELDLEILGKLVVGIGEVSEITQIPVRKIRYWEDKGIIRSLTEEEGKNRRYDY
RNIKKILLIKELLDEGFTLEAAAKKVENRIATVNSVFEQLTKFKKTKS

>MBD98870.1 - *Verrucomicrobia bacterium*
MDSDPSKLYYTIGEVSRMFDVNTSLIRFWEKEFDILKPKKNKKGNRLFRKQDIDNLKLIYHLVKVRGFTL
DGAKKKLRENKEETVNQLQMVDSLKKIRSFLIGLKEEL

>NLB64605.1 - *Fibrobacter sp.*
MFYTIGEVADMFQVNASLIRFWEKEFPIIKPKKNAKGNRLFRPEDIKNLQIIYHLVKEQGMTLKGAKKRI
QNNKEGTQQNIEIINKLTAIKQKLLAIQEHL

>WP_012458164.1 - *Porphyromonas gingivalis*
MKKQKQGKLFYSISEVARMFDLPGSTLRFWEKEFPALKPRTSGGGTRRYTAKDIEMVRLIHHLTKEKGLT
LAGTKQALKNDYDGTTRREEVISRLKEIRQELCDIRDAIDQWERKNMY

>QPN47327.1 - *Priestia aryabhattai*
MLEPSHNDELPPIPGKRYFTIGEVSELCAVKPHVLRYWEQEFPQLNPVKRRGNRRYYQRQDVLMIRQIRA
LLYDQGFTIGGARLRLSSDEVKDESSQYKQLIRQMIVELEDVLVVLKK

>CJK57227.1 - *Streptococcus pneumoniae*
MLEPSHNDELPSIPGKRYFTIGEVSELCAVKPHVLRYWEQEFPQLNPVKRRGNRRYYQRQDVLMIRQIRA
LLYDQGFTIGGARQRMSGDEARDDTTQYKQLIRQMISELEEVLQVLRK

>NNL76386.1 - *Desulfobacterales* bacterium
MQKPYQTKIPDKLYFKIGEVSKISGLPSHVLRFWETEFKKINPRRTGSGQRSYTPKDVAIILEIKHLLHE
KKFTIEGARKHLGAKSRQDSAATEQFLTKLKAELISIRDLLDSN

>MBN1547652.1 - *Syntrophaceae* bacterium
MNVTLPDKAYYRIGEVSKYLKVEPYVIRFWETEFKILKPVRASSGHRLYRQKDVEALVLIRQLLYEQRFT
ISGAKQYLAGMARETTAAETDTDRRRLVEIKKELTGIRNLIVKNK

>WP_063719089.1 - *Leptolyngbya valderiana*
MLDPGSNQELPTIPAKRYFTIGEVSELCAVKPHVLRYWEQEFPQLKPVKRRGNRRYYQRHDVLMIRQIRS
LLHEQGFTILGARQRLEGEKAQDDVSMSQQLIRQLRTELEDILHVLRR

>TSD66519.1 - *Inquilinus sp.*
MPYKEREISKMYYTMGEVSAMFDVNQSLIRFYEKEFDVLQPKKNKKGNRYFTPEDIENFKIIFHLIRDKG
YTLQGAKDHLKNNMGESRDNQRVINSLENMKKFLLEVRDQL

>WP_071838703.1 - *Tolypothrix campylonemoides*
MLDPGSNRELPPIPAKRYFTIGEVSELCDVKPHVLRYWETEFPMLSPVKRRGNRRYYQRHEVLMVRQIRG
LLYEQGYTIGGARQRLEGESGKTEAALSAQIIKQVRVELEEVLQLLRR

>REH36807.1 - *Paraperlucidibaca baekdonensis*
MSMSITATNATLPEIPNKRYLTIGEVSELCDVKCHVLRYWEQEFPSLKPVKRRGNRRYYQRTDVQLIRRI
RELLYFQGYTIQGARSQLAESKTGPLTSPSTQAVLAPAASKSPAENAPLQRVIDEMLSDLEQIEQLLRQD
A

>SCZ05944.1 - *Acinetobacter baumannii*
MLEPSHNDELPPIPGKRYFTIGEVSDLCAVKPHVLRYWEQEFPQLNPVKRRGNRRYYQRQDVLMIRQIRA
LLYDQGFTIGGARQRLSGDEAKDDVTQYKQLIRQMIAELEEVLLVLKK

>WP_063492348.1 - *Bordetella trematum*
MTKPETTATLPPIPAKRYFTIGEVSELCGVKPHVLRYWEQEFTQLKPVKRRGNRRYYQHHEVLLIRRIRS
LLYEQGFTISGARNRLGDARDNPQDSAEAAVRLSPAELQSLRNELDDISAMLAQAIGSPASA

>MQY66210.1 - *Clostridia* bacterium
MQIPDKNFFSIGEASKITGVKSYILRYWESEFKLLRPARRESGHRKYTRKDLEVIGEIKELLYERRFSIA
GAKRHLLDDRKNKKEQLKLDWGKDSAAISALKDTKKEIKEILKLLEK

>MBT0814463.1 - *Octadecabacter sp.*
MHPLADKRYYSIGEVAKAFEVNTSLIRFWENTFDILQPKKNAKGNRKFTPEDIKNLQLIYHLVKEKGYTL
EGAKAHLKVSKAKTADQLELVKRLESVREELLKIKEQL
